# Applications of synthetic biology in biomedicine

**DOI:** 10.1186/s43556-026-00545-x

**Published:** 2026-08-19

**Authors:** Chen-Xuan Li, Zi-Xuan Liu, Yu-Fan Lin, Zi-Heng Wang, Yi-Min Yang, Si-Yi Feng, Yan-Han Sun, Xiao-Tong Zhang, Wei Fu, Yu-Fan Ding, Chun-Chen Gao, Hong-Yan Qin, Jun-Long Zhao

**Affiliations:** 1 State Key Laboratory of Holistic Integrative Management of Gastrointestinal Cancers, Department of Medical Genetics and Developmental Biology, Fourth Military Medical University, Xi’an, 710032 China; 2 Basic Medical College, Fourth Military Medical University, Xi’an, 710032 China; 3 Department of Gastroenterology, 925th Hospital of PLA Joint Logistics Support Force, No. 67, Huanghe Road, Guiyang, 510009 China

**Keywords:** Synthetic biology, Biomedicine, Gene circuit, Cell therapy, Clinical translation

## Abstract

Based on the principles of engineering reconstruction and programmable design, synthetic biology is driving a paradigm shift in biomedical diagnosis and therapy from conventional models toward intelligent and precision medicine. By constructing artificial genetic circuits, functional cells, and biomaterial systems both in vitro and in vivo, synthetic biology markedly enhances diagnostic sensitivity, therapeutic targeting, and clinical benefit. In recent years, with the maturation of key technologies such as DNA synthesis and assembly, computational modeling, gene editing, RNA regulation, and protein engineering, synthetic biology has spawned numerous applications with potential for clinical translation in fields such as early screening for pathogens and tumors, programmable cellular immunotherapies, intelligent life-based therapies, and the manufacture of medical biomaterials. Nevertheless, current synthetic biology systems still face critical bottlenecks such as insufficient targeting and editing precision in vivo, poor functional stability of gene circuits, pronounced immunogenicity risks, high manufacturing costs, and lagging ethical and regulatory frameworks. This review systematically summarizes progress in foundational tools and key supporting technologies of synthetic biology, highlights innovative strategies and clinical value in biosensors, cell therapy, living therapeutics, and smart biomaterials, and provides an in‑depth comparison of different chassis cells, delivery vectors, and regulatory circuits in terms of disease suitability, safety, and translational efficiency. The artificial intelligence (AI)-enabled component design, closed-loop intelligent regulation, off‑the‑shelf universal cells, and multimodal theranostic platforms are also discussed. This review offers a systematic framework from technical principles to clinical translation and provides theoretical support and technical guidance for developing next-generation synthetic biology-based diagnostic and therapeutic strategies.

## Introduction

Synthetic biology is an interdisciplinary engineering discipline. By integrating modular design, standardized assembly, and programmable regulation, it enables the creation of biological functions beyond the reach of natural evolution [1, 2]. Synthetic biology is a transformative force in 21st‑century life sciences. It integrates engineering principles with biological knowledge, moving beyond the mere understanding of living systems toward building and even reshaping them [3]. In the highly complex and urgently needed application domain of biomedicine, synthetic biology is systematically rewriting the logic of traditional medical practice from diagnostics and therapeutics to the manufacturing of medical biomaterials.

The impact of synthetic biology on biomedicine has been particularly profound. It plays a key role in innovative disease mechanism research, diagnostic technology development, drug discovery paradigms, and precision medicine strategies. By integrating artificial genetic circuits [4], biosensors [5], and effector elements [6] into cells or biomaterials, researchers have created intelligent, controllable, and accessible therapeutic agents that can sense pathological signals, perform complex logic operations, and release therapeutic molecules on demand [7]. These innovations open new possibilities for overcoming medical challenges that have long been difficult to address with conventional therapies, including tumors, autoimmune diseases, metabolic disorders, refractory infections, and rare genetic diseases.

Nevertheless, synthetic biology still faces numerous core challenges on the path from laboratory proof-of-concept to widespread clinical translation. First, a mature, efficient, and standardized set of basic tools and technology systems is essential to support the development of the field, covering DNA synthesis and assembly, computational design and modeling, and gene editing and regulation [8, 9]. Second, translating these technologies effectively into specific diagnostic and therapeutic applications, while ensuring their targeting, safety, and stability in the complex in vivo environment, is a central focus of current research [10]. In addition, high research and production costs, potential biosafety risks, lagging ethical and regulatory frameworks, and uncertainties in clinical translation pathways have become key bottlenecks hindering industrialization.

**Fig. 1:**
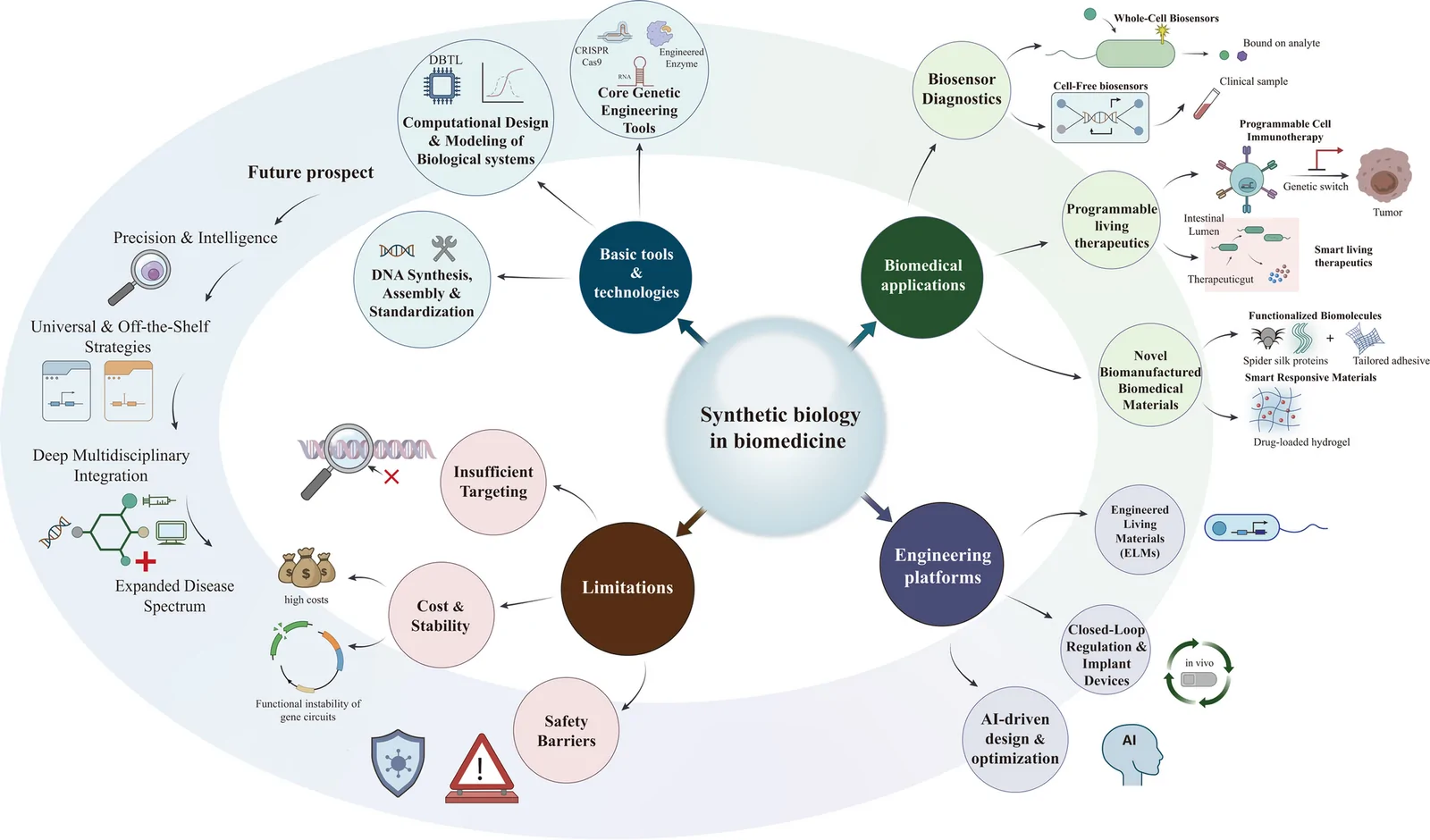
Applications of synthetic biology in biomedicine. This figure depicts the field as a cohesive ecosystem in which foundational technologies, including DNA synthesis, computational modeling, and gene and protein engineering, which enable the creation of biosensors, living therapeutics, and advanced biomaterials. These applications are in turn supported by delivery platforms and intelligent control systems that enhance their in vivo performance and safety. However, clinical translation remains constrained by key challenges such as insufficient targeting, high costs, system instability, and immunogenicity. Addressing these barriers through precision-controlled design, off-the-shelf manufacturing, and interdisciplinary integration will ultimately position synthetic biology as a cornerstone of next-generation medicine

Against this background, this review aims to systematically survey the basic tools and core technologies of synthetic biology in biomedicine, summarize recent progress in diagnostic, therapeutic, and biomanufacturing applications, and analyze the major limitations and challenges in current clinical translation (Fig. 1). On this basis, we further discuss future directions for synthetic biology, exploring how multidisciplinary integration, artificial intelligence empowerment, and regulatory innovation can help transform synthetic biology from a “disruptive technology” into a “clinically reliable solution”, ultimately providing a systematic reference for achieving precision medicine and improving human health and well-being.

## Basic tools and technologies

### DNA synthesis, assembly and standardization

#### DNA synthesis

DNA synthesis constitutes a foundational technology within synthetic biology, providing the essential genetic material required for the construction of artificial biological systems. Over the past several decades, advancements in DNA synthesis have progressed from the production of short oligonucleotide chains to the synthesis of longer gene segments, culminating in the capability to synthesize entire genomes. Presently, the predominant methods for DNA synthesis encompass chemical and enzymatic approaches [8]. The chemical synthesis approach primarily relies on the solid-phase phosphoramidite triester method, established by Carruthers’ team in 1987 [11]. However, as sequence length increases, the cost rises exponentially, and chemical impurities may be introduced, making it difficult to meet the needs for long fragments and large-scale libraries in synthetic biology [12].

Enzymatic DNA synthesis (EDS) represents a distinct approach from traditional chemical methods by employing template-independent polymerases, notably terminal deoxynucleotidyl transferase (TdT), to catalytically elongate DNA strands within aqueous environments. This strategy circumvents the stepwise coupling inefficiencies that limit the scalable production of long oligonucleotides via standard solid-phase chemical synthesis [13]. Early research demonstrated that terminal deoxynucleotidyl transferase (TdT) could add dNTPs to a DNA strand's 3′-hydroxyl end in the absence of a template strand, allowing for de novo DNA synthesis [14]. In recent years, with advances in enzyme engineering and reversible protecting group technology, Lu [15] achieved accurate sequence construction by adding a terminator at the 3′ end, ensuring that only one nucleotide is added per reaction step [16, 17]. Concurrently, recent studies have successfully obtained engineered TdT enzymes with improved thermostability and developed reversible 3′-dNTP protecting groups compatible with TdT, further enhancing enzymatic coupling efficiency and enabling cyclic solid-phase template-free DNA synthesis [15]. Due to its immunity to the coupling efficiency decline that affects chemical synthesis as chain length increases, EDS possesses a distinct advantage in the synthesis of lengthy DNA strands (e.g., > 200 nt) [15], and is applicable to high-fidelity long-strand DNA and epigenetically modified DNA (e.g., methylated DNA) [18]. It offers a new route for precise sequence synthesis of gene therapy vectors such as adeno-associated viruse (AAV) and lentivirus. Moreover, EDS possesses significant high-throughput capabilities, with novel technologies (e.g., electrochemically cleavable TdT conjugates) facilitating parallel synthesis on microelectrode arrays, establishing a basis for extensive industrial applications [19]. Although this method excels in long-sequence and high-purity synthesis, its efficiency is limited by enzyme specificity and reaction conditions [20]. In recent years, hybrid synthesis strategies that integrate chemical and enzymatic approaches have arisen, seeking to optimize efficiency and cost-effectiveness, thereby advancing DNA synthesis technology towards longer sequences, increased throughput, reduced expenses, and enhanced automation [21].

#### DNA assembly technology and standardization

DNA assembly technology plays a crucial role in the integration of multiple small DNA fragments into longer sequences or complex genetic constructs. The earliest strategies for assembling DNA fragments trace their origins to the discovery of restriction endonucleases and DNA ligase, whose complementary functionalities enabled the first generation of recombinant DNA experiments. In this classical approach, insert and vector are separately digested and subsequently covalently joined via ligase [22]. However, this method requires specific restriction sites, is labor-intensive, and frequently results in the incorporation of undesirable “scar” sequences [23]. To achieve standardized assembly of biological parts, researchers developed the BglBrick system in an attempt to standardize biological components (BioBricks) so that DNA parts from different laboratories could be used compatibly. In 2003, the Knight team at MIT introduced the BioBrick standard for the physical assembly of biological components, overcoming the constraints of part integration and facilitating the directed assembly of various BioBrick elements. Moreover, the resulting fusion parts themselves retain BioBrick properties, allowing continued free combination with other similar parts [24]. Researchers engineered fixed restriction sites (EcoRI, XbaI, SpeI, and PstI) at the termini of DNA fragments and employed compatible cohesive ends produced by isocaudomers (XbaI and SpeI) to facilitate "modular" splicing of DNA elements. New restriction sites are preserved post-splicing, facilitating boundless cascading. This method offers high standardization and simple operation, suitable for constructing basic gene circuits. However, the residual scar sequences from restriction digestion affect protein expression and circuit function and prevent seamless splicing [25]. To address the scar sequence problem in BioBrick assembly, Golden Gate assembly technology emerged. Established by Marillonnet’s team in 2008, it uses Type IIS restriction enzymes (e.g., BsaI, BsmBI), whose recognition sites are separate from their cleavage sites, generating 4-bp asymmetric cohesive ends after digestion. Through the meticulous design of complimentary terminal sequences, multiple DNA fragments can be accurately and seamlessly assembled in a single-tube process with over 90% efficiency and devoid of scar sequences, rendering it ideal for the efficient construction of intricate gene circuits and multigene metabolic pathways [26].

Recently, homology-based assembly techniques that do not depend on specific sequences have been advanced. Gibson et al. established by Gibson's team in 2009, is the predominant technology for the seamless integration of extensive fragments [27, 28]. Additionally, the GoldBricks method has been introduced, which incorporates Type IIS restriction sites into traditional BioBrick components. This innovation combines the simplicity and modularity of the BioBrick system with the efficiency and speed characteristic of Golden Gate assembly. Notably, this approach enhances assembly efficiency and presents distinct advantages over Golden Gate cloning, including support for operon-based functional designs and reductions in both library construction scale and manual labor [29]. Beyond the above methods, other emerging assembly techniques continue to appear, including Uracil-Specific Excision Reagent (USER) assembly (using uracil-excision enzymes), DATEL assembly (using thermostable nucleases), and enzyme-free cloning (EFC) [30], which simplify reaction systems and reduce costs. Furthermore, the integration of automated liquid handling systems and microfluidic technologies is significantly increasing assembly throughput, providing robust platforms for the large-scale fabrication of complex biological systems [31].

Through the implementation of standardized systems for genetic components and communal part libraries, synthetic biology can facilitate the "plug-and-play" integration of biological elements, address the “black box” challenge in biological system design, and offer standardized toolkits for biomedical applications. DNA standardization technology establishes the technical framework for synthetic biology via “standard part libraries” and “standard assembly techniques” [24]. In about 2003, MIT's iGEM competition and the BioBricks Foundation officially introduced the "BioBricks" standard, marking the inaugural effort to modularize and standardize the design of biological components, including promoters, genes, and terminators. Consequently, the iGEM Registry of Standard Biological Parts, the largest public DNA part repository globally, was created, encompassing tens of thousands of standardized components [32]. The ongoing optimization and cohesive use of these technologies are markedly expediting the transition of synthetic biology from laboratory study to practical implementation.

### Computational design and modeling of biological systems

The primary characteristic that differentiates synthetic biology from conventional biology is the use of rational design informed by computational models, as opposed to empirical trial and error [33]. Biological systems are intricate, nonlinear, and unpredictable [34], rendering direct experimentation wasteful and expensive. Through the integration of computational design, mathematical modeling, system simulation, and artificial intelligence-driven optimization, researchers are able to predict system behaviors and optimize component parameters, therefore realizing an engineering methodology of “simulate first, build later”. This significantly enhances the success rate in the design of intricate biological systems, including intelligent therapeutic cells, metabolic drug production pathways, and precision diagnostic circuits.

#### Design-build-test-Learning (DBTL) cycle

The DBTL cycle is a fundamental engineering framework in synthetic biology. It is fundamentally an iterative closed-loop engineering approach that attains continual self-optimization by relaying knowledge from the “Learn” phase to the “Design” phase [35]. This methodology transforms traditional intuition- and experience-driven biology into a field with the predictability and programmability of electronic engineering, greatly accelerating the development of new biological systems such as metabolic pathways and gene circuits [36]. Design: Use computational tools to design genetic components, circuits, and systems. Build: Through synthetic DNA fragments, self-assembly, chassis cell modification, and cell-free system construction, translate designs into physical biological entities, completing gene editing, strain construction, and microbial community assembly. Test: Perform high-throughput, multidimensional testing of the constructed biological system to obtain actual performance data and verify whether the design meets specifications. Learn: Utilize experimental data to enhance the preliminary model, conduct system analysis, modify parameters, optimize the design, extract performance rules, and guide the subsequent design iteration, so creating a closed iterative loop. Ongoing enhancement of protein efficacy can be attained over numerous iterative cycles [37].

#### Mathematical modeling of biological systems: from qualitative description to quantitative prediction

In contemporary synthetic and computational biology, mathematical modeling serves as the fundamental link for converting biological systems into engineering systems. Rigorous mathematical models transform biological systems into computable frameworks that facilitate quantitative functional predictions [38]. Based on scale and method, modeling can be divided into component-level, circuit-level, and cell-population-level.

A component is the most basic functional unit of a biological system. Component-level modeling abstracts biologically meaningful functional units (e.g., promoters, ribonucleases, protein degradation tags) into quantifiable mathematical objects using thermodynamic and kinetic models [39, 40]. For example, based on RNA polymerase binding free energy, promoter transcription efficiency (RPU, relative promoter unit) can be predicted, and promoter sequences optimized to meet expression requirements [39].

A gene circuit integrates many components to perform sensing, logical processing, and signal output. The essence of modeling is to delineate regulatory linkages and dynamic reactions among components. Ordinary differential equation (ODE) models are prevalent deterministic models that characterize dynamic variations in mRNA and protein concentrations over time through ODEs, appropriate for high-concentration, low-noise systems. An ODE model of a tumor necrosis factor (TNF)-inducible circuit can forecast reaction time, expression intensity, and saturation threshold across varying TNF concentrations [41]. Stochastic models (e.g., Gillespie algorithm) account for molecular randomness (noise) inherent in biological systems, suitable for low-molecule-number systems (e.g., gene expression in single cells). They can predict noise levels, stability, and probabilistic behaviors. For instance, researchers have used stochastic models to design gene lysis circuits that reduce viral expression noise and improve consistency of tumor cell lysis [42]. Boolean logic models simplify gene circuits into binary “on/off” states, suitable for rapid construction of complex gene regulatory networks and analysis of their steady-state or periodic behavior; they are computationally efficient and especially useful when precise dynamic parameters are unavailable [43].

Cell population modeling extends single-cell models to the macroscopic scale, aiming to predict and control the dynamic behavior of large cell populations across space and time [44]. Modeling of cell populations requires a comprehensive approach that integrates the simulation of intracellular genetic network activities, the characterization of heterotypic and homotypic cell–cell signaling (encompassing both synergistic and antagonistic interactions), and the assessment of microenvironmental parameters such as substrate supply and waste product levels, all of which jointly orchestrate emergent population-level phenotypes [45]. Agent-based modeling (ABM) treats each cell as an independent agent with individual properties, simulating highly heterogeneous population behaviors such as proliferation, migration, signal transduction, and death. ABM is well-suited for simulating cutting-edge population behaviors in synthetic biology, such as quorum-sensing spatiotemporal synchronization and population computing, and it can capture how individual differences are amplified or suppressed in collective behaviors [46].

#### Computational design tools and platforms

Synthetic biology has developed a computational design toolchain covering the entire process from component design and circuit simulation to metabolic optimization and automated DBTL, providing convenient and efficient design platforms for biomedical applications.

##### Component and sequence design tools

Generating DNA sequences efficiently and accurately is the first priority before performing complex circuit or genome design. A core tool is Genetic Design Automation (GenoCAD), a “grammar”-based gene design tool. It abstracts DNA sequences as “words” and biological elements (promoters, RBS, genes, etc.) as “words”, designing complex gene networks as “sentences”. Through “autocompletion” and “semantic checking”, GenoCAD can rapidly generate DNA sequences that conform to biological norms, offering great flexibility while reducing manual design errors [47].

##### Gene circuit design and simulation tools

After designing elements, it is necessary to verify the dynamic behavior of the circuits. Core tools include iBioSim, and the main standard is Synthetic Biology Open Language (SBOL) [48]. iBioSim supports modeling from component level to circuit level, based on Boolean logic and ODE models, and can simulate spatiotemporal dynamics of gene expression. iBioSim facilitates the quantitative forecasting of synthetic circuit performance by converting abstract “logic gates” into operational “mathematical equations” using formal computational models. SBOL works as the “assembly language” of synthetic biology, offering a standardized data format for delineating component sequences, functionalities, and interactions. Through SBOL, designed circuits can be seamlessly shared among different laboratories and software platforms, greatly facilitating design reproducibility and modularity [49].

##### Metabolic network tools

When research involves multiple metabolic pathways or an entire cellular chassis, higher-order computational models are required. Common core tools include Bioscrape (BioSynthetic Circuit Analysis, Simulation and Design) and Constraint-Based Reconstruction and Analysis (COBRA). Bioscrape is a computational framework built on Python that combines efficient stochastic simulation capabilities, accelerated via Cython with the ability to model both intracellular noise at the single-cell level and diffusional coupling at the population scale [50]. It combines stochastic simulation algorithms (SSA) and ODE models to simulate the evolution of synthetic circuits within a cell population [51]. COBRA is primarily used for constructing and analyzing metabolic networks; it predicts cellular metabolic behavior under different environments through flux balance analysis (FBA). In metabolic engineering, COBRA helps designers predict changes in product yield after gene knockout or overexpression and is a core tool for metabolic pathway optimization [52].

#### Automation and database

To manage the vast component libraries and experimental data, unified databases and automation platforms are needed. Common examples include SynBioTools and JBEI-ICE [53]. SynBioTools is a one-stop search and selection platform for synthetic biology tools, aggregating nearly a thousand tools from around the world to help researchers quickly find needed software resources [54]. JBEI-ICE is an open-source biological part registry platform. It records not only sequence information but also part function, experimental results, and version history. It supports an Application programming interface (API) interface and can communicate directly with other design software (e.g., iBioSim) to enable automated data flow [53].

### Core enabling tools for genetic manipulation

The cross-disciplinary innovation of synthetic biology and precision medicine relies on the collaborative application of four core technologies: gene editing, RNA regulation, protein engineering, and genetic circuit construction [55]. These technologies provide powerful tools for understanding life mechanisms and treating diseases, respectively addressing genome modification, post-transcriptional regulation, protein function remodeling, and cell behavior programming.

#### Gene editing technology

Gene editing technology, represented by CRISPR-Cas9, has become the tool of choice for constructing synthetic genomes and precision cell therapies due to its efficiency, precision, and programmability [56]. The Cas9-sgRNA ribonucleoprotein complex functions as a programmable endonuclease, wherein the single-guide RNA specifies genomic target recognition and directs the Cas9 protein to catalyze site-specific double-stranded DNA cleavage through a mechanism that requires protospacer-adjacent motif (PAM) recognition for activation [57]. Cells then repair the breaks via non-homologous end joining (NHEJ) or via homology-directed repair (HDR) to insert new sequences, enabling precise genome editing [9]. In immune cell engineering, CRISPR-Cas9 is widely used to knock out immune checkpoint genes such as PD-1 and CTLA-4, relieving T cell immunosuppression and enhancing anti-tumor activity [58]. In tumor cells, this technology can target oncogenes such as Kirsten rat sarcoma viral oncogene homolog (KRAS) and Myelocytomatosis oncogene (MYC) to inhibit proliferation [59], or restore tumor suppressor genes such as p53 and PTEN [60], reshaping tumor cell growth regulatory networks. Additionally, CRISPR-Cas9 can be used to modify the tumor microenvironment, suppress tumor progression, and promote immunotherapy [60, 61].

#### RNA regulation technology

RNA regulation technologies, including RNA interference (RNAi), ribozyme regulation, antisense oligonucleotides (ASOs), and adenosine deaminase acting on RNA (ADAR) editing systems, enable reversible and controllable regulation of cellular functions by precisely intervening in gene expression at the post-transcriptional level [62, 63]. RNA interference (RNAi) is a common regulatory mechanism in eukaryotic cells that utilizes small interfering RNA (siRNA) or short hairpin RNA (shRNA) to activate the RNA-induced silencing complex (RISC), which specifically recognizes and degrades target mRNA, thereby effectively silencing gene expression [64]. Ribozymes are catalytically active RNA molecules; the hammerhead ribozyme (HHR) can recognize and bind target RNA complementary to its binding site, specifically cleaving target mRNA or inhibiting translation, achieving gene silencing. They offer high specificity, low immunogenicity, and reusability, providing new tools for disease treatment [65].

Antisense oligonucleotides (ASOs) are short, single-stranded deoxyribonucleotide chains, typically 10 to 30 bases in length, that recognize and bind to complementary sequences on target mRNAs via Watson–Crick base pairing. Upon cellular internalization, the ASO-mRNA duplex recruits RNase H, a ubiquitously expressed endonuclease that specifically cleaves the RNA strand within the heteroduplex, thereby suppressing translation of the target transcript [66]. ASOs have four main mechanisms: (i) targeting pre-mRNA to inhibit capping and polyadenylation, thereby blocking maturation; (ii) regulating alternative splicing via exon skipping or inclusion; (iii) activating RNase H to degrade hybrid-bound target RNA and inhibit translation; and (iv) binding to genomic DNA to form triple helices, inducing DNA damage and blocking transcription [67]. For example, the lipid droplet protein ADRP mediates hepatic steatosis. ASO treatment targeting ADRP enhances hepatic insulin action, increases IRS1, IRS2, and Akt phosphorylation, and reduces gluconeogenic enzymes and PKCε [68].

The ADAR editing system is an RNA-targeted editing tool that recruits endogenous ADAR proteins. It catalyzes the deamination of adenosine (A) to inosine (I) in double-stranded RNA; inosine is recognized as guanosine (G) during translation, enabling site-directed editing or expression regulation of target genes [69]. For example, ADAR editing can repair disease-causing mutations (e.g., in TP53 and IDUA) to restore normal protein function [70], or edit immune-related genes (e.g., ADAR1) to regulate RNA sensing pathways, enhance innate immune responses in tumor cells, and improve immunotherapy outcomes [71]. Compared to DNA editing, RNA editing offers advantages of reversibility, no permanent genome changes, and low immunogenicity, making it particularly suitable for treatment scenarios requiring time-dependent regulation [72].

#### Protein engineering

Protein engineering provides a variety of molecular tools for precision medicine by artificially designing and modifying the amino acid sequences of natural proteins, reshaping their structure and function, and constructing functional proteins with novel properties. In receptor modification, the affinity, specificity, and signal transduction efficiency of natural receptors can be optimized through site-directed mutagenesis and domain fusion [73]. For example, amino acid mutations in the extracellular single-chain variable fragment (scFv) of a chimeric antigen receptor (CAR) can enhance binding affinity to tumor antigens [74]. Optimizing the combination of transmembrane and intracellular costimulatory domains can improve CAR-T cell proliferation, cytokine release, and in vivo persistence [75]. The artificial alteration of proteases and transcription factors aims to regulate essential intracellular signaling networks. For example, mutating the active center of proteases can yield substrate-specific tool enzymes for precise cleavage of specific signaling molecules [76]. On this basis, the construction of chimeric receptors, cleavable proteins, and synthetic signaling molecules further expands the application scenarios of protein engineering. For instance, chimeric receptors cleavable by specific proteases in the tumor microenvironment enable tumor-specific activation of CAR-T cells and reduce off-target toxicity [77]. Synthetic signaling molecules can be engineered to accurately modulate intracellular signaling networks, granting cells distinct functional responses [78].

## Biomedical applications of synthetic biology

### Biosensor-based diagnostic applications

In the field of synthetic biology, a biosensor constitutes a recognition and analysis platform that converts biochemical reactions (e.g., antigen–antibody specific binding, DNA hybridization, cell metabolism) into detectable electrical, optical, or thermal signals. Its defining feature is the use of synthetic biology methods to integrate biological components with engineering logic, implementing programmable and modular systems [79], thereby achieving greater flexibility, precision, programmability, and standardization than traditional biosensors for monitoring a wide range of biological and environmental stimuli.

A typical synthetic biology biosensor generally consists of two functional modules: (i) the biological recognition element, responsible for specific recognition of target molecules (e.g., pathogens, drug-resistance genes, biomarkers) [80], often comprising modified proteins (e.g., CRISPR-Cas, Argonaute proteins) or nucleic acids (e.g., aptamers, riboswitches); and (ii) the signal transduction and output element (reporter system), which converts the recognition event into a detectable signal, most commonly through reporter genes such as fluorescent proteins (e.g., GFP, RFP), luciferase, or pigment proteins [81].

Based on the environment in which the synthetic biosensor’s genetic circuit operates, biosensors can be divided into whole-cell biosensors and cell-free biosensors [82]. The following sections introduce frontier progress of synthetic biosensors in disease diagnosis from these two categories.

#### Whole-Cell Biosensors (WCBs)

WCBs transform engineered living cells into programmable living detectors by embedding genetic circuits. They convert disease-related molecules into detectable signals, enabling specific and efficient disease diagnosis. They also offer advantages of self-repair, self-replication, and low cultivation cost [83].

In cancer diagnosis, traditional methods suffer from invasiveness, pain, low sensitivity, and low specificity, whereas synthetic biosensors can address these issues. Brasino et al. engineered Lactiplantibacillus plantarum into an in vivo lung cancer biosensor. After colonizing the lung, the bacteria secreted nanoluciferase in response to model peptides from lung cancer cell lines, which was detected in urine, enabling non-invasive in vivo tumor screening [84]. Cooper et al. designed an engineered bacterial sensor to detect tumor DNA in colorectal cancer and other tumors. Utilizing the inherent capacity of Acinetobacter baylyi to assimilate extracellular free DNA, they engineered specific guide sequences to correspond with targets (e.g., the G12D mutation in KRAS) and integrated them with an innovative biosensing approach, CRISPR-CATCH, to accurately detect particular cancer mutations and induce reporter gene expression, thereby facilitating the visual identification of specific tumor DNA sequences [85, 86].

In addition, transgenic bacterial biosensors have shown great potential for non-invasive gastrointestinal disease diagnosis due to their ability to efficiently detect and respond to specific disease signals in the gut. Yang et al. designed an antiterminator microbial whole-cell biosensor (MWCB) based on Bacteroides thetaiotaomicron that exhibited strong responses to nitrate and nitrite in the inflammatory environment in vivo, with response intensity significantly correlated with colitis inflammation level, enabling detection and diagnosis of colitis [87]. Buss et al. developed a “diagnostic yogurt” based on probiotic E. coli Nissle 1917; after ingestion, it transiently populated the gastrointestinal tract, sensed inflammation biomarkers, and produced ultrasound-detectable acoustic contrast via expression of gas vesicles, allowing non-invasive imaging diagnosis of inflammation by whole-cell biosensors [88]. The MagGel-BS bacterial biosensing platform developed by Xu et al. used heme-hypersensitive strains to detect gastrointestinal bleeding within 20 min [89].

In the field of metabolic disease diagnosis, Gao et al. proposed a microbial whole-cell sensing strategy based on Bacillus subtilis spores, achieving highly sensitive detection via glucose-induced spore germination in body fluids to generate self-powered electrical signals, offering a new approach for non-invasive wearable diabetes monitoring [90].

In recent years, WCBs have been widely used for detecting disease-related target molecules. Leveraging the properties of viable cells and combining them with optimized genetic circuit design and delivery technologies, the clinical translation prospects of WCBs for accurate disease diagnosis are highly promising.

#### Cell-free biosensors (CFBs)

CFBs circumvent the limitations of living cells by employing cell-extracted transcription-translation processes to provide in vitro detection platforms. This removes the cell membrane barrier, significantly reduces response time, and provides exceptional storage stability and biosafety owing to their non-living characteristics. Therefore, CFBs have shown unique value for target detection scenarios requiring rapid, convenient, safe, and sensitive analysis, particularly in point-of-care diagnostics and resource-limited settings [91–93].

Viral diseases pose serious threats to human health, driving urgent need for rapid virus detection strategies. CFBs can meet this requirement, enabling in vitro, rapid, and convenient detection. Pardee et al. developed a paper-based virus detection platform based on a cell-free expression system, in which core components were freeze-dried onto filter paper and stably stored at room temperature. When a water sample containing the target analyte was dropped onto the paper, the cell-free expression reaction was activated, and a toehold switch specifically recognized viral nucleic acid and simultaneously activated reporter gene expression to generate a detectable signal. In subsequent experiments, researchers successfully applied cell-free paper biosensors to screen multiple viruses, including Ebola virus [94].

Furthermore, CFBs for bacterial nucleic acid detection have matured considerably. Park et al. proposed a modular CRISPR-Cas-based platform that converts Cas-mediated site-specific collateral cleavage into protein output [95]. Using Bacillus anthracis and E. coli O157:H7 as models, they developed CRIVER to simultaneously detect the 16S rRNA of B. anthracis and the species-specific ecf1 locus of E. coli O157:H7 in a dual-channel workflow by coupling Cas13a-mediated RNA recognition and Cas12a-mediated DNA recognition, achieving a detection limit as low as 100 CFU/mL, demonstrating its ability to detect sensitive (pathogenic) microorganisms [95].

In addition to the most common optical signals, cell-free biosensors can also output via electrochemical signals. Mousavi et al. combined a genetic circuit with an electrode interface to design a scalable system that cuts specific DNA sequences in solution and generates an electrochemical signal when the released DNA strands are captured on the surface of nanostructured microelectrodes, enabling detection of target nucleic acids by the presence or absence of an electrical current. The researchers successfully applied this to multiplex detection of colistin resistance genes, demonstrating the powerful performance of this new electrochemical cell-free biosensor [96] (Table 1).

**Table 1:** Synthetic biology-derived biosensors for disease detection

Platform type	Delivery method	Bio-sensing element	Target disease	Functional performance	Refs
Lactiplantibacillus plantarum WCFS1	Oropharyngeal aspiration	SspKR two-component system coupled to urinary nLuc secretion	Lung cancer	Non-invasive in vivo tumor peptide detection; pulmonary residence without colonization	[84]
Acinetobacter baylyi	Rectal enema	CATCH system (CRISPR-HGT) with reporter gene activation	CRC/KRAS mutations	Single-nucleotide specificity; in situ tumor DNA detection; stool-based readout	[86]
Bacteroides thetaiotaomicron VPI-5482	Oral gavage	NasR antiterminator-regulated nLuc expression	Colitis/Nitrate, Nitrite	Gut-commensal chassis; in vivo inflammation sensing correlated with histopathology	[87]
Escherichia coli Nissle 1917	Oral gavage; GI colonization	ThsSR/TtrSR two-component coupled to gas vesicle-mediated ultrasound contrast	IBD/Thiosulfate, Tetrathionateprotein A	First noninvasive in situ ultrasound imaging of GI inflammation; molecular-to-anatomical mapping	[88]
Bacillus subtilis (Endospore-based MFC)	Paper-based MFC	Glucose-triggered spore germination coupled to electrical output	Diabetes/Glucose (sweat)	Self-powered, enzyme-free sensing; exceptional shelf-life stability	[90]
Cell-free system (Paper-based)	Freeze-dried, rehydrated	Toehold switch-regulated colorimetric (LacZ) output	Antibiotic resistance; Ebola	Room-temperature stable; rapid prototyping; field-deployable, naked-eye diagnostics	[94]
Cell-free system (CRIVER)	On-chip, dual-channel	Programmed Cas12/13 collateral cleavage coupled to protein reporter synthesis	Pathogen DNA/RNA	CRISPR recognition converted to protein output; dual genus/species identification from single sample	[95]
Cell-free system (Paper-based, Electrochemical)	Freeze-dried, rehydrated	Switch-regulated electrochemical signal via enzyme reporters	MCR genes; Viral RNA	Multiplexed, orthogonal detection; direct gene circuit-to-electrode interface	[96]

### Programmable living therapeutics

Synthetic biology enables researchers to design therapeutic strategies with specific recognition and precise intervention through modularization and programming of biological systems [97]. From programmable cell therapies (e.g., CAR-T, CAR-M, SynNotch-based) to smart living therapeutics, and from simple drug delivery to targeted delivery, synthetic biology is continuously expanding the boundaries of disease treatment.

#### Programmable cell immunotherapy

Synthetic biology dramatically improves the safety and precision of cell therapies by embedding engineered logic-computing capabilities into immune cells, granting them the ability to sense multiple signals, achieve specific activation, and execute complex regulatory responses [98]. In recent years, programmable cellular immunotherapy has advanced along two main lines: the maturation of logic gating technologies and the diversification of effector cell types [99].

Logic gating technology is a core technical pathway for precision immune cell engineering, endowing programmable immune cells with more accurate decision-making capabilities. By integrating Boolean logic such as AND, OR, and NOT into CARs [100], CAR-T cells can precisely distinguish target identity under multiple antigen input conditions, thereby overcoming the “on-target, off-tumor” toxicity and tumor antigen heterogeneity faced by traditional CAR-T cells in solid tumors [7]. The synthetic Notch (SynNotch) receptor is one of the most representative engineering platforms in this field, providing a route for flexibly shaping personalized and inducible immune cell responses [101]. Roybal et al. engineered SynNotch receptors linked to the expression of downstream effector genes, allowing T cells to react to specific antigen signals and precisely execute predefined therapeutic response programs (e.g., cytokine secretion, CAR expression), significantly enhancing the programmable capabilities of immune cell therapy. The traditional SynNotch-based two-layer gating design involves sensing antigen A via the SynNotch receptor to induce CAR expression, and then the CAR recognizes antigen B to trigger killing. Rommel et al. further developed a single-vector SynNotch system. By optimizing packaging and transduction of large transgenes in T cells, they successfully constructed dual-target logic-gated T cells targeting HER2⁺MSLN⁺ ovarian tumors, which showed better targeting selectivity and anti-tumor efficacy than traditional dual-vector schemes both in vitro and in vivo [102].

At the same time, effector cell types in programmable cellular immunotherapy are expanding from T cells to broader lineages. Although CAR-T cells have achieved milestone breakthroughs in hematologic malignancies, their application in solid tumors is limited by poor tumor infiltration, strong immunosuppression in the tumor microenvironment (TME), and tumor antigen heterogeneity [103]. In contrast, CAR-M (CAR-macrophage) has emerged as a novel platform for solid tumor therapy due to its excellent tumor chemotaxis and invasion, strong phagocytic function, and unique TME-remodeling capability. CAR-M can not only directly target tumor cells through CAR signaling but also activate adaptive immune responses via a “phagocytosis-presentation-activation” cascade, producing synergistic effects with immune checkpoint blockade. At the engineering level, CAR-M has evolved from early construction to logic-gated circuits and in vivo in situ generation strategies based on lipid nanoparticles [104]. In 2025, Reiss et al. reported the first-in-human phase I clinical trial of CT-0508 CAR-M therapy in patients with HER2-overexpressing advanced solid tumors [105]. This represents not only the first clinical validation of the safety, tolerability, and feasibility of CAR-M therapy but also opens the way for macrophage-based immunotherapy.

#### Smart living therapeutics

Living therapeutics are created by modular assembly of biosensors, logic gates, and effector elements via synthetic biology, transforming common microorganisms into programmable therapeutic platforms and opening new avenues for disease treatment. With modular genetic circuits, engineered bacteria can sense disease-related signals, perform logical operations, and release therapeutic drugs at disease sites. Currently, EcN and Lactococcus lactis have become major strains for therapeutic applications, being reprogrammed for treatment of inflammatory bowel disease (IBD), metabolic diseases such as phenylketonuria, and cancer therapy [106].

**Fig. 2:**
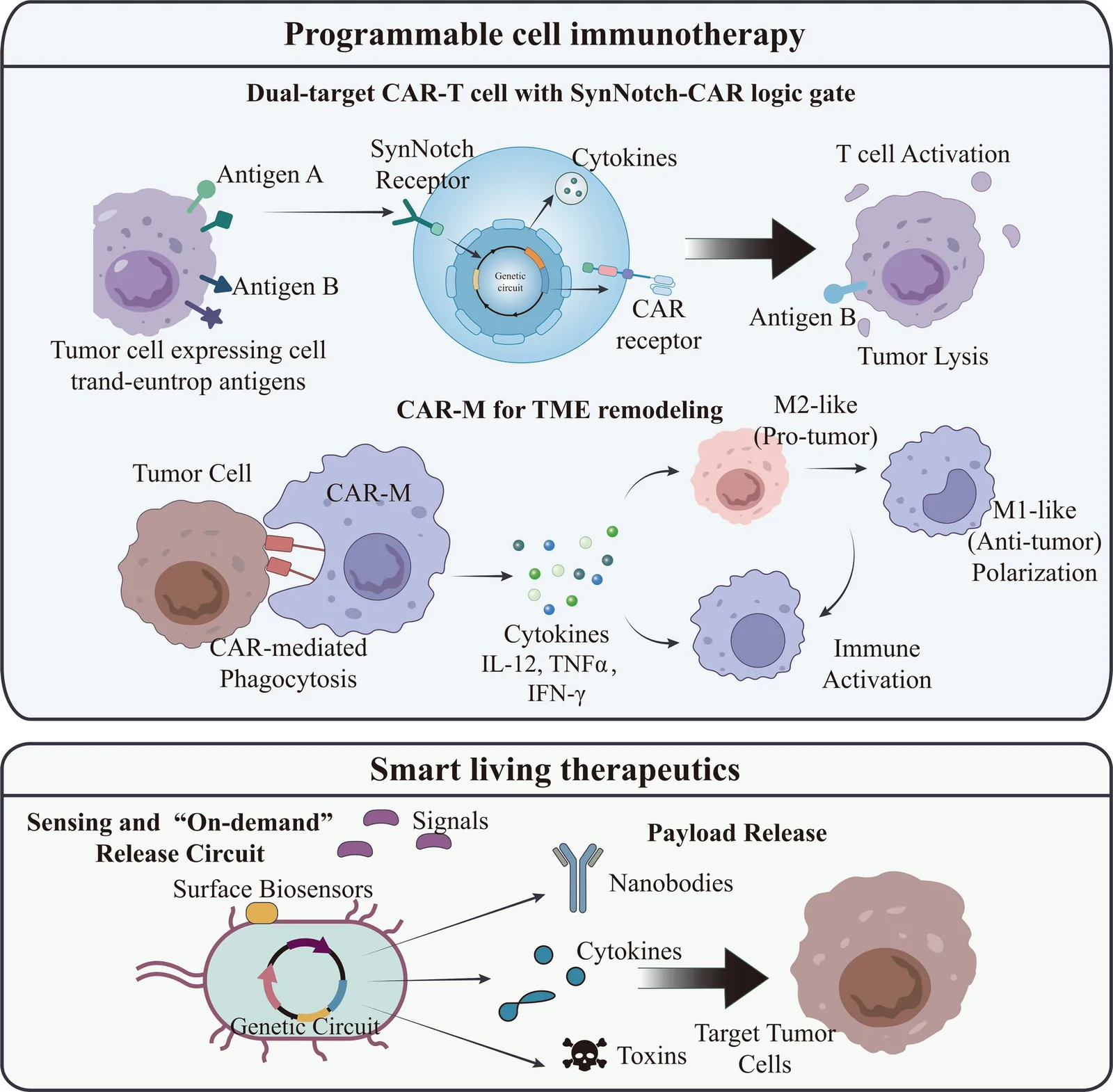
Frontier advances in the field of therapeutics. This figure summarizes the core design strategies of Programmable cell immunotherapy and smart living therapeutics. Dual-targeting CAR-T cells and SynNotch logic gates are shown on the upper side. Tumor cells express both antigens A and B, and SynNotch logic gates allow precise targeting by recognizing both antigens. The mid-upper part is CAR-M for TME remodeling. CAR-M recognizes and engulfs tumor cells through CAR-mediated phagocytosis, releases cytokines such as IL-12, TNF-α, IFN-γ, and drives the polarization of M2-like (pro-tumor) macrophages to M1-like (anti-tumor) phenotype, and then activates immunity, lyses tumors, activates T cells, and forms immune responses. On the bottom side, smart living therapeutics contains a "sense and release on demand loop", surface biosensors recognize signals, and genetic circuits control the release of payloads (nanoantibodies, cytokines) to target and kill tumor cells

Utilizing modular assembly and multi-tiered regulation, intelligently designed bacteria can accurately detect small molecules, tumor microenvironment signals, and inflammatory mediators, subsequently activating treatment protocols under defined conditions. This enables “on-demand treatment” and “conditional release”, thereby overcoming the constraints of continuous drug exposure and passive distribution inherent in conventional therapies. In tumor immune regulation, multi-input logic circuits that recognize tumor-specific signals enable engineered bacteria to selectively express immunomodulatory molecules. In tumor immune regulation, multi-input logic circuits that detect tumor-specific signals allow engineered bacteria to selectively produce immunomodulatory chemicals or lethal agents within the tumor microenvironment, therefore amplifying anti-tumor actions while minimizing systemic adverse effects [107] (Fig. 2). Deng et al. modified Escherichia coli Nissle 1917 (EcN) to serve as an IL-12 expression platform, regulated by both hypoxia and quorum sensing, and employed engineered dendritic cells (eDCs) to enhance bacterial delivery and offer immunological assistance. The system effectively suppressed tumor growth and metastasis and prolonged survival [108].

### The development of new biomanufacturing innovative medical materials

Through targeted modification of microbial genomes via synthetic biology, engineered cells can efficiently and controllably synthesize medical materials with specific structures and functions. In this context, functional biomolecules and intelligent responsive materials represent the cutting edge of novel biomedical materials, transforming the material basis for tissue engineering, wound healing, and drug delivery.

#### Functionalized biomacromolecules

Spider silk protein has long been regarded as an ideal natural biomaterial due to its ultra-high tensile strength, excellent elasticity, and outstanding biocompatibility [109]. It offers unique advantages in tissue engineering scaffolds, drug delivery carriers, surgical sutures, wound dressings, bandages, and medical implant coatings. However, large-scale production has historically been a formidable bottleneck. Advances in synthetic biology have broken this limitation, making efficient production and functional engineering of recombinant spider silk proteins possible. Poddar et al. proposed that by expressing recombinant spider silk proteins in heterologous hosts such as E. coli, yeast, and plants, the natural limitations of spider farming can be avoided, and silk protein sequences can be customized via genetic engineering to endow them with new bioactivities [110].

Bacterial cellulose (BC) is a nano-cellulose material synthesized by specific microbial strains during fermentation. It features an ultrafine nanofiber network, excellent mechanical strength, high water-holding capacity, and natural biocompatibility. Compared to plant cellulose, it has higher chemical purity and contains no lignin or hemicellulose, making it particularly suitable for biomedical applications [111]. Kulshrestha et al. methodically elucidated the synthesis mechanism and commercial applications of BC, demonstrating that both in situ and exogenous modification procedures can significantly boost BC's efficacy in tissue engineering, drug administration, and wound repair [112]. At the clinical translation level, Zhong et al. reported an engineered hemostatic dressing (T-BC) based on a cellulose-binding domain with recombinant human thrombin anchored to the BC matrix. T-BC exhibited excellent immediate hemostatic ability and a significant pro-healing effect in a rat deep partial-thickness burn model. Transcriptome analysis indicated that it regulates multiple signaling pathways involved in angiogenesis, inflammation resolution, and extracellular matrix remodeling [113].

#### Smart responsive materials

An important direction for next-generation medical materials is to incorporate the “smart” properties of synthetic biology into material design. Unlike static inert scaffolds, smart responsive materials are designed to sense specific environmental signals and respond according to pre-programmed instructions, transitioning from passive filling to active interaction in applications such as drug delivery and tissue repair [114].

Xu et al. developed a biomimetic polymer membrane-coated insulin crystal system, in which both glucose and β-hydroxybutyrate-dual-sensitive microdomains are integrated into the nanofilm. Under normal physiological conditions, the microdomains are uncharged and nanopore channels are narrow, preventing insulin release. Under hyperglycemic and ketonemic conditions, the microdomains convert high concentrations of glucose and β-hydroxybutyrate into a negative potential on the membrane surface, causing nanopores to expand and insulin to be released rapidly. In a murine model of type 1 diabetes, the system sustained normoglycemia for over one month, exemplifying a significant advancement of intelligent responsive materials in the prolonged management of metabolic disorders [115].

Nawaz et al. pointed out that pH is the primary response factor affecting the delivery performance of cellulose-based complexes for anti-cancer drugs [116]. Therefore, tumor microenvironment-specific pH-responsive design is an urgent strategy for smart anti-cancer drug delivery systems. Lee et al. developed a pH-responsive platform (pDCs) based on DNA nanostructure-functionalized cellulose nanocrystals. pDCs achieve stable drug encapsulation at physiological pH. Under acidic conditions in the tumor microenvironment, the i-motif on the pDC surface undergoes a conformational change, weakening the interaction between DNA nanostructures and Cellulose nanocrystals (CNC), triggering release of the drug encapsulated inside CNC and enabling precision drug delivery to cancer cells [117] (Table 2).

**Table 2:** Summary of biofabrication advancing medical materials

Platform Name	Material Composition	Application	Functional Payload	Key Results	Refs
Bacterial Cellulose (BC)	BC + chitosan, gelatin, HA, AgNPs, GO, etc	Tissue engineering, wound dressings, drug delivery	*In-situ*/*ex-situ* modification for tailored properties	Comprehensive review of BC modification strategies, tissue engineering applications, and commercial products	[112]
Thrombin-Anchored BC (T-BC)	Thrombin-CBD fusion protein + BC	Hemostatic burn wound dressing	CBD-mediated thrombin anchoring for rapid hemostasis and tissue repair	60 s hemostasis in rat liver; 88.7% wound closure (day 5); anti-inflammatory and angiogenic mechanisms confirmed	[113]
Thrombin- Intelligent Insulin Crystal (i-crystal)	Insulin crystal + glucose/ketone-responsive polymeric membrane	Long-acting, self-regulated T1D therapy	Membrane potential-gated insulin release triggered by hyperglycemia or ketonemia	38-day normoglycemia (mice, single injection); 533 h (minipigs); dual-responsive pulsatile release validated	[115]
Polymeric DNA-decorated CNCs (pDCs)	Polymeric DNA (aptamer & i-motif) + cationic CNCs	Targeted intracellular cancer therapy	Aptamer-mediated cancer targeting; pH-responsive i-motif triggers Dox release	eightfold cancer-selective uptake; 63.4% apoptosis in HeLa cells; minimal off-target toxicity	[117]
Probiotic Living Materials (PLMs)	Engineered probiotics + scaffolds	Multifunctional therapeutics	Disease biomarker-sensing genetic circuits for on-demand therapeutic production	Reviewed design strategies and applications; identified challenges in genetic tools, biosafety, and translation	[118]
Biohybrid Living Material (BHLM)	L. acidophilus + TiO₂ NPs + PNIPAM hydrogel	Burn wound dressing	TiO₂ NP-induced oxidative hormesis enhances probiotic growth and H₂O₂ synthesis	twofold bacterial growth increase; pathogen viability reduced to 16.9–25.1%; rapid wound closure (0.14 cm^2^ at day 21)	[119]
Recombinant Spider Silk Proteins (rSSPs)	rSSPs (fibers, films, hydrogels, particles)	Tissue engineering, drug delivery, biosensors	Genetic fusion of RGD peptides or functional motifs to spidroin sequences	Reviewed rSSP production and biomedical applications; highlighted RGD-modified scaffolds and energy devices	[120]

## Engineering platforms for therapeutic delivery and control

Traditional immunotherapy generally suffers from pain points such as short survival of living vectors, high off-target risk, and uncontrollable immune responses [121]. The iterative upgrading of multifunctional support platforms and smart delivery systems addresses these issues. This part examines engineered living materials, in vivo closed-loop intelligent control, implantable devices, and AI-driven design technologies, methodically assessing the fundamental mechanisms, application contexts, and developmental prospects of each technology.

### Engineered Living Materials (ELMs) as interactive therapeutic platforms

ELMs are the fundamental outputs of the cross-integration between synthetic biomedicine and functional biomaterials [118]. Focused on the directing alteration of living cells, ELMs are integrated with three-dimensional biological scaffolds that exhibit intrinsic biological activity and structural integrity, enabling sustained in situ therapeutic function delivery [122, 123]. They transcend the constraints of standard biomaterials, which are characterized as “non-living and non-responsive” by naturally integrating the functionality of living cells with material stability, thereby providing a novel carrier option for immunotherapy [124]. Compared to traditional carriers and scaffolds, the core advantage of ELMs lies in their ability to preserve the natural physiological activity of living cells, offering programmable bioactivity, longer functional lifespan, and enhanced environmental adaptability, enabling integration of lesion identification, pathological evaluation, targeted therapy, and tissue repair [125]. This advantage is realized through the selection of appropriate chassis cells. Conventional selections of engineered immune cells [126], mesenchymal stem cells [127], and engineered microbes [128] as chassis cells can address the limitations of quick apoptosis and poor targeting associated with natural cells, rendering them appropriate for diverse complex pathological conditions. Loebinger et al. conducted a study suggest that bone marrow-derived mesenchymal stem cells (MSCs) can home to and integrate into tumor tissue, and that MSC-based strategies are steadily advancing toward preclinical stages [129].

#### Core standardized design principles

The compliant large-scale construction of ELMs follows a trinity design principle of “programmable cell function, highly adaptive material interface, and microenvironment responsiveness”. The synergistic support of these three elements builds the foundation for long-term, safe, and efficient adaptation of ELMs [123]. Cell function reconstruction is the core, material interface adaptation is the foundation, and microenvironment adaptation is the guarantee; together they constitute a standardized construction system [130].

First, modular reprogramming of cell function in living chassis. By using synthetic biology tools such as the SynNotch system and logic gate regulatory circuits, researchers can directionally reconstruct the cell signal recognition, transduction, and output, customizing differentiated therapeutic functions to meet individualized diagnosis and treatment needs [131, 132]. In practice, this can specifically enhance solid tumor infiltration, reverse the tumor microenvironment, and secrete anti-inflammatory factors in a gradient manner to adapt to different disease scenarios [133]. Second, optimization of interface biocompatibility of composite biomaterials. Medical-grade degradable and low-immunogenicity substrates are optimized to build a three-dimensional support framework that encapsulates and protects the living units, prolongs their survival, and creates a stable active environment [122, 123]. Shen et al. utilized medical-grade type I collagen gel (low immunogenicity, biodegradable) to fabricate a three-dimensional porous scaffold via crosslinking and solidification molding. This scaffold encapsulated neural stem cells, successfully prolonging cell survival and establishing a stable microenvironment for neural stem cells. Results show that the three-dimensional collagen gel culture system is superior to suspension culture in the proliferation, differentiation and process outgrowth of neural stem cells [134]. Third, intelligent adaptive responses to the pathological microenvironment in vivo. Relying on synthetic gene sensing circuits, characteristic signals such as tumor hypoxia, pH imbalance, and inflammatory factors can be captured, and downstream gene circuits linked to automatically start or stop therapeutic functions, forming a “sensing-transduction-therapy” loop [135]. Typical applications include the in situ activation of antitumor therapy via hypoxia-responsive elements based on Hypoxia-Inducible Factors (HIFs), which function as master regulators of oxygen homeostasis in all metazoan species, as well as the adaptive regulation of anti-inflammatory cytokine secretion through inflammation-inducible circuits, ensuring therapeutic precision and safety. Currently, rapid progress is being made in elucidating the homeostatic roles of HIFs in various physiological systems, determining the pathological consequences of their dysregulation in chronic diseases, and exploring their potential as therapeutic targets [136].

#### Classification and application of ELMs in immunotherapy

Engineered immune cells combined with living targeting materials: These materials are based on CAR-T/CAR-NK cells, macrophages, and dendritic cells, and can be combined with biological scaffolds to compensate for the short survival and weak targeting of natural immune cells. They are the most mature ELMs in tumor immunotherapy [137, 138].

The hydrogel and microcapsule encapsulation avoid immune clearance, prolong cell survival, and enhance solid tumor infiltration, thereby reducing off-target toxicity [139, 140]. Combined with SynNotch circuits and hypoxia-activated circuits, they achieve in situ tumor killing and normal tissue silencing, with safety performance superior to traditional CAR-T therapy [133, 141–143]. Beyond CAR-T/CAR-NK platforms, macrophages represent another important chassis for ELM-based immunotherapy. ELMs can directionally edit macrophages to an M1 anti-tumor phenotype in the tumor microenvironment or adapt to autoimmune scenarios by secreting anti-inflammatory factors with a high safety threshold [144–146]. In dendritic cell-targeted therapy and tumor vaccine systems, tumor-specific antigens can be loaded onto biomimetic scaffolds to achieve excellent lymph node targeting capacity, effectively target dendritic cells in lymph nodes, enhance antigen presentation by inhibiting the mevalonate pathway, and promote cytotoxic T cell infiltration, thereby inhibiting tumorigenesis and attenuating tumor progression, while simultaneously achieving long-term immune surveillance, thus accomplishing the dual effects of tumor suppression and relapse prevention [147].

ELMs are also applied to colonization of living microbiota. Safe probiotics and non-pathogenic E. coli are preferred as chassis. After directional modification, they can attach to and colonize the mucosa, constructing a low-cost, long-term mucosal immune protection system [148, 149]. The engineered materials can express tumor antigens and immune adjuvants; after oral administration, they colonize the intestine, activate anti-tumor immunity of the intestinal mucosa, and balance the gut flora. They can also serve as living drug factories to synthesize and secrete immune regulatory factors, suitable for scenarios such as intestinal inflammation and superficial tumors, natural tumor-targeting and probiotic bacteria have been engineered for controlled and sustained delivery of anticancer agents into the tumor microenvironment, with excellent preclinical results [150]. Yue et al. genetically engineered Escherichia coli to express a specific tumor antigen fused with cell lysis protein A under the control of an arabinose-inducible promoter, resulting in an oral tumor vaccine, which successfully activated anti-tumor immune responses and induced immunological memory, significantly suppressing tumor growth [151]. Ge et al. genetically engineered non-pathogenic Escherichia coli to secrete the adhesion protein CP43K and continuously release the intestinal barrier repair factor TFF3 upon detecting bleeding signals, thereby improving weight recovery, reversing colon shortening, reducing intestinal inflammation, and repairing the mucosal barrier [152].

#### Advantages and clinical translation potential of ELMs

Compared to traditional immunotherapy technologies, ELMs offer three core advantages. First, the scaffold isolates and protects living units from immune clearance, prolonging their survival and reducing administration frequency and cost [140]; second, enabled by surface modification and microenvironment sensing, ELMs reduced off-target risk [153]; third, a single component realizes multifunctional adaptation, simplifying treatment regimens [154]. Currently, ELMs have achieved remarkable results in melanoma, pancreatic cancer, rheumatoid arthritis, and other chronic diseases, with some categories entering early clinical trials [155].

Engineered biomaterials are revolutionizing immunotherapy through three core advantages: their protective scaffold structure can prolong the duration of drug efficacy and reduce the cost of drug delivery. Nie et al. employed an innovative 3D-printed immune scaffold to anchor CAR-Macrophages (CAR-M), enabling sustained long-term antitumor effects with a single implantation of a small number of cells, thereby significantly reducing therapeutic material consumption and preparation costs; their dual mechanisms of surface modification and microenvironment sensing ensure precise targeting while significantly minimizing off-target side effects [156]. Zheng et al. engineered bifunctional outer membrane vesicles that leverage dual mechanisms to achieve lesion-directed enrichment, substantially avoiding non-specific damage. Experimental results demonstrate that the tumor-targeting platform enhances the antitumor activity of CAR-T cells both in vitro and in vivo, and promotes CAR-T cell expansion by ameliorating the tumor microenvironment. BROAD-CAR also facilitates the in situ antigenic modification of solid tumors, and mediates CAR-induced lysis of antigen-heterogeneous and antigen-negative tumors, inhibiting tumor recurrence and metastasis in breast cancer mouse models [154].

### Closed-loop regulation and implantable devices for in vivo therapeutic control

Traditional immunotherapy lacks real-time sensing and dynamic regulation mechanisms [157], making it prone to cytokine storms [158], immune cell exhaustion [159], and other safety hazards that restrict clinical scale-up. The synthetic gene regulatory circuits establish a closed loop of sensing-regulation-intervention, realizing intelligent and safe immunotherapy and becoming core supports for technology translation.

#### The in vivo feedback regulation closed-loop system

Negative-feedback closed-loop regulation aims to precisely curb immune overactivation. Its core function is “strength control”, targeting risks such as cytokine storms and immune-related adverse events. It builds a closed loop of “sensing-suppression-reset”, automatically initiating suppression programs when the immune response exceeds a safe threshold [137]. Three main technical pathways exist: (i) Cytokine secretion negative feedback: implant cytokine-specific sensing elements to capture changes in pro-inflammatory factor concentrations (e.g., IL-6, IFN-γ); when a threshold is exceeded, down-regulate pro-inflammatory factor expression and up-regulate anti-inflammatory factor secretion, thereby alleviating cytokine storms [160]. (ii) T cell activation negative feedback: implant a dephosphorylase expression circuit in engineered T cells; when TCR is continuously activated or activation markers are too high, inhibit signal transduction, reduce excessive proliferation, delay immune exhaustion, and prolong cell survival [161]. (iii) Immune checkpoint independent expression: implant conditional expression loops for PD-L1, CTLA-4, etc., increasing their expression during immune response spikes to form an intrinsic “brake”, controlling treatment intensity, evading immune reactions, and avoiding excessive immunosuppression from exogenous inhibitors [162].

Positive-feedback closed-loop regulation aims to enhance immune response efficacy. Targeting limitations such as insufficient immune response and cell exhaustion, it builds a closed loop of “sensing-amplification-reinforcement,” activating signal amplification programs when response intensity falls below a threshold to prolong the treatment period [137]. Two core pathways exist: (i) Cytokine autocrine positive feedback: implant autocrine circuits for pro-proliferative factors [163]; when immune cell activity decreases, automatically secrete factors to achieve self-activation and proliferation, prolong survival, and strengthen anti-tumor responses. (ii) Antigen recognition-amplified positive feedback: relying on the SynNotch system, construct dual-antigen recognition circuits; after recognizing the first tumor antigen, initiate expression of a second CAR to enhance killing efficiency and overcome tumor heterogeneous drug resistance [141].

#### Inducible regulatory system for precise control of immunotherapy

Inducible regulatory systems enable on-demand start/stop and remote regulation of treatment, triggering gene circuits via external controllable signals to improve treatment flexibility and safety [164]. These systems include small molecule induction and physical signal induction [165]. Small compounds, by traditional drug delivery, are appropriate for extensive clinical application [164]. Mainstream inducers encompass doxycycline and rapamycin, both of which have shown clinical validation for safety [166]. Moreover, induction switch systems utilizing small-molecule antiviral agents (e.g., simeprevir) offer a safer mechanism for on-demand initiation and cessation of CAR-T therapy [167]. Most small molecule drugs sense G-protein-coupled-receptors (GPCRs). The modified GPCRs respond only to artificial inducers, allowing rapid start/stop to manage toxicity risks [168].

Physical signal induction systems use non-invasive physical triggers such as light, oxygen, ultrasound, and magnetic fields, offering non-invasiveness, good tissue penetrability, no drug residues, suitability for deep tumor treatment and other special sites, precision positioning control, and low off-target toxicity [169]. However, they require specialized equipment and represent a direction for high-end individualized treatment. The light-controlled gene circuit systems use near-infrared light (for deep tumors) or blue light (for superficial lesions) to trigger circuits via implanted light-sensitive proteins, achieving high spatiotemporal precision and avoiding normal tissue damage [170]. The implanted hypoxia response elements [171], activating only in tumor hypoxic regions, enable autonomous lesion identification and in situ treatment. Focused ultrasound can penetrate deep tissues and induce localized thermal effects, thereby enabling the activation of transgenes, thus enhancing the targeting and regulatory efficiency for deep-seated tumors [172].

#### Implantable integrated devices for closed-loop therapy

Implantable integrated devices are the physical carriers of closed-loop control systems, integrating four modules. The living functional units are equipped with modified immune cells and regulatory circuits to achieve targeted therapy; biomaterial carrier encapsulates and protects the functional unit, enabling uniform and sustained factor release; microelectronic module monitors immune responses and microenvironment in real time and receives remote regulatory signals; biodegradable components ensure non-toxic degradation after treatment completion, improving patient experience. Minimally invasively implanted into the body, an implantable miniaturized device using electrode-embedded optical fibers with both local delivery and measurement capabilities is developed over the course of a few weeks, thereby enabling integrated whole-process control. Their controllability and integration are far superior to traditional systems; they are small, degradable, and do not require secondary surgical removal [173].

**Fig. 3:**
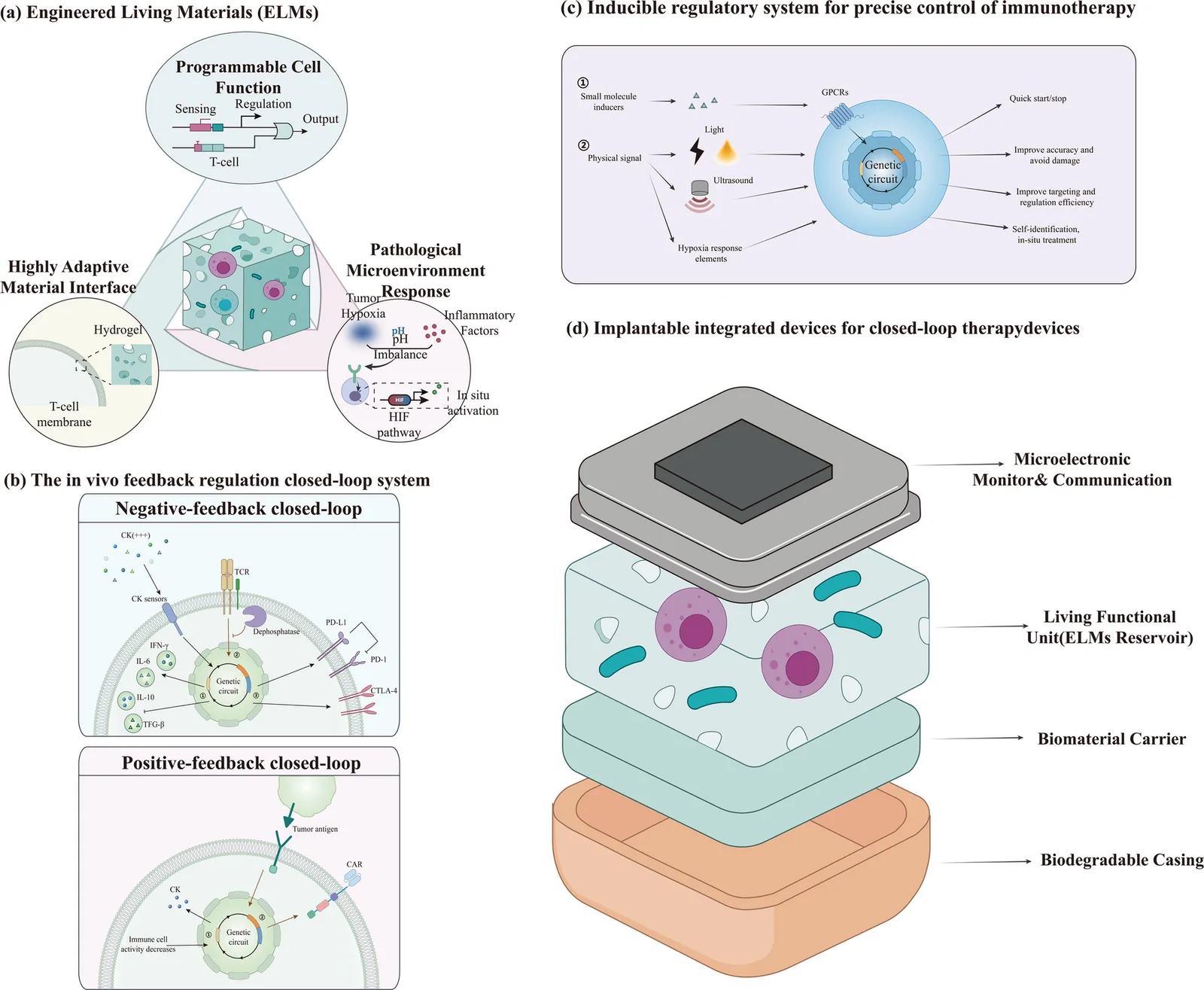
Support platform and delivery system. **a** Engineered living materials (ELMs) integrate programmable cells with scaffolds to sense signals, process information, and execute targeted therapy in situ. **b** Closed-loop feedback regulation mechanisms, comprising both negative and positive feedback loops, enable dynamic adjustment of therapeutic activity in response to real-time physiological signals, thereby maintaining treatment within a safe and effective window. **c** Inducible systems enable external control via small molecules or physical cues for on-demand regulation. **d** Implantable devices combine sensors, living units, biomaterials, and biodegradable shells for real-time monitoring and intervention at disease sites

These implantable devices are currently adapted to different medical application scenarios. Localized intelligent tumor treatment device achieves real-time monitoring of microenvironment and cytokines, automatically initiates CAR-T killing function, and avoids Cytokine Release Syndrome (CRS) risk [137]. Implantation near inflammatory lesions helps to monitor autoimmune factors in real time, and avoids systemic side effects. Madhvapathy et al. implanted an implantable temperature sensor into a mouse model of Crohn's disease-like ileitis to detect changes in the concentrations of stress hormones and inflammatory cytokines in the blood for early detection and intervention of inflammatory bowel disease [174]. Infection defense device implanted at susceptible mucosal sites releases anti-infective substances on demand, and strengthens mucosal immunity. Rahim et al. thermally anchored symbiotic microbiota onto the surface of titanium implants, which could modulate macrophage polarization to control infection and facilitate tissue repair. This strategy endows implants for infection-prone mucosal sites such as the oral cavity with anti-infection and immunomodulatory functions [175] (Fig. 3).

### Artificial intelligence-driven design and optimization of synthetic biology

Through core capabilities such as deep learning and protein structure prediction, artificial intelligence (AI) technology enables “de novo design, automatic optimization, and individualized adaptation” of functional components, driving synthetic immunotherapy towards intelligence and personalization.

#### AI-driven de novo protein design

The core functional vehicles of synthetic immunotherapy are proteins, whose spatial conformation and function directly determine therapeutic safety and efficacy. The application of artificial intelligence to structural biology has transformed protein design from a conceptual challenge into a practical approach for creating new-to-nature proteins, freeing it from natural templates [176]. Currently, mainstream AI design tools have formed a complete ecosystem. RFdiffusion generates atomically precise protein three-dimensional structures, meeting antigen-receptor binding requirements [177]. AlphaFold2 accurately predicts protein structures, identifies active sites, and guides modification and optimization [178]. ProteinMPNN designs highly stable and active amino acid sequences based on structure [179]. DiffPepBuilder, based on diffusion models, enables precise construction of target-specific de novo peptide conjugates [180]. These three methods together form a complete technical system for protein design [181].

AI design tools can assist in the design of high-affinity immune receptors. AI de novo designs CAR/TCR antigen-binding domains, combined with AlphaFold2 optimization, significantly improving binding affinity and specificity, thereby reducing off-target toxicity. Based on the BindCraft framework, one-stop de novo efficient design of functional protein binders can be achieved [182]. Based on deep learning, de novo precise design of cytokine or enzyme mimics, such as IL-2 and IL-15, shows higher activity by optimizing sequence structure, prolonging half-life, enhancing activity, and reducing immunogenicity [144]. An AI-optimized IL-15 variant showed significantly prolonged half-life and enhanced bioactivity. AI-designed Cas9 variants increase specificity, reduce off-target risk, and improve editing efficiency [183].

#### AI-driven automated design of artificial gene circuits

Gene circuits are the core of synthetic immunotherapy with high design complexity. AI-driven design achieves full-process of design-screen ADDIN EN.CITE vision and reliability. Current mainstream AI design platforms have been industrially applied. Cello 2.0 automatically designs and virtually verifies gene circuits according to functional requirements, screens optimal designs, and avoids module conflicts [184]. SynBioSuite integrates omics data for more personalized circuit design tailored to individuals. GECCO learns the shape of a performance landscape and iteratively navigate the design space toward an optimal circuit. This strategy allows the joint optimization of both circuit architecture and parameters, and enables parametric sweeps to assess circuit robustness to perturbations [185] (Table 3).

**Table 3:** AI-driven design of biomedical synthetic systems

Platform	**Core Principle**	**Application**	**Key Innovation**	**Key Result**	**Refs**
RFdiffusion	Diffusion Model; RoseTTAFold backbone	Protein backbone generation; Binder design	Fine-tuning structure prediction network for denoising	Atomic-accuracy binders; Diverse symmetric oligomers	[177]
AlphaFold2	Evoformer; Structure Module	Protein structure prediction	End-to-end learning; Iterative refinement via recycling	Atomic-accuracy prediction at CASP14; pLDDT confidence metric	[178]
BindCraft	Backpropagation through AF2; Hallucination	One-shot binder design; Functional site targeting	Target flexibility; Integration of MPNNsol optimization	Nanomolar affinity binders; 10–100% experimental success rate	[182]
BayesOpt for Circuits	Bayesian Optimization	Multiscale genetic circuit optimization	Joint optimization of integer architecture and continuous parameters	Rapid convergence; Outperforms genetic algorithm	[185]
ProteinMPNN	Message-Passing Neural Network	Protein sequence design; Structure rescue	Order-agnostic autoregressive decoding; Multi-chain coupling	High solubility & thermostability; Outperforms Rosetta	[186]
Helical Peptide Binders	RFdiffusion (Partial Diffusion); Hallucination	Picomolar-affinity peptide binder design	Binder design to flexible targets; Partial diffusion for optimization	Picomolar binders; Binder-based biosensor & LC–MS/MS enrichment	[187]
PBPK-ML Nano-Framework	PBPK Modeling; Machine Learning	In vitro & in vivo nanotoxicity prediction	Integrating PBPK-derived exposure metrics into ML framework	High PR-AUC & recall; Identified concentration as key toxicity predictor	[188]

AI design platforms are used in personalized gene circuit design. Combining patient tumor heterogeneity and immune status data, AI-designed gene circuits satisfied “one patient, one circuit,” significantly improving treatment efficacy compared to generic designs. AI design is also applicable to complex circuit optimization. It enables virtual simulation of the in vivo environment, optimization of module parameters, and correction of defects such as response delays [189]. As a result, the response speed of AI-optimized negative feedback loops is improved, and the incidence of CRS is reduced.

#### AI-driven intelligent screening and optimization of delivery vectors

Delivery vectors directly determine the efficiency and safety of functional component delivery. Traditional methods for optimizing the selection of delivery materials and component ratios rely on manual trial and error and experimental validation with long cycles and low efficiency. AI-driven selection and optimization technologies, based on big data and molecular simulation, enable rapid, accurate optimization screening and individualized adaptation of vectors, sharply increasing research and development (R&D) efficiency [190, 191].

With the aid of AI tools, the advantages, functions, and defects of existing carriers can be systematically organized—a task too enormous for manual screening to accomplish. By mining the structure-performance relationships of different carriers and combining them with molecular simulation-driven optimization, it becomes possible to simulate vector behavior in vivo, identify defects, and optimize composition and surface modifications to improve targeting and stability [188]. Current targeted delivery biocarriers mainly include biomaterial scaffolds, nanocarriers, and viral vectors. AI optimizes biomaterial scaffolds in terms of composition, pore size, and surface modifications to improve encapsulation protection and targeting of immune cells [192]. Meanwhile, AI screens targeted modification molecules on different nanocarriers to enhance tumor penetration and stability [193]. For the selection of viral serotypes, AI optimizes immunogenicity and off-target sites to improve gene delivery accuracy [194].

## Limitations of biomedical applications based on synthetic biology

### Insufficient targeting of in vivo editing

The core advantage of synthetic biology in biomedicine lies in providing “programmability” and “precise regulatory capability” to therapeutic systems [195]. However, when these genetic circuits are transferred from the laboratory environment to the complex in vivo environment, the precise targeting ability shown in vitro often decreases significantly in vivo [196]. As Yin et al. have summarized, key obstacles include off-target effects, immunotoxicity caused by engineered T cells, and the limited capacity of therapeutic systems to adapt to dynamic disease progression. These barriers can be further categorized into three progressive levels: off-target editing at the molecular level, on-target/off-tumor toxicity at the cellular level, and loss of delivery specificity at the tissue level [10].

#### Off-target effects in CRISPR gene editing

Gene-editing tools, especially the CRISPR-Cas system, still face serious challenges. The targeted recognition of the CRISPR-Cas9 system relies on complementary base pairing between the guide RNA and target DNA, but the gRNA can tolerate a certain degree of sequence mismatch. Fu et al. demonstrated that even single or double mismatches can be tolerated, and they identified off-target sites harboring up to five mismatches that were mutagenized at frequencies comparable to on-target sites, indicating that Cas9 may still cleave at genomic sites even in the presence of 3–5 mismatched bases [197].

A further concern is that assessing off-target effects is itself a technically unresolved challenge. Existing fidelity assessment strategies often capture limited aspects of editing outcomes and do not fully account for context-dependent biochemical, epigenetic, and cellular heterogeneity that can influence therapeutic safety and efficacy, making it difficult to accurately predict real editing behavior and off-target risk [198]. This assessment uncertainty directly translates into regulatory approval dilemmas.

#### On-target/off-tumor toxicity at the cellular level

Taking CAR-T therapy as an example, the root cause is that tumor-associated antigens (TAAs) are not unique to tumors; most TAAs are also expressed to varying degrees in normal tissues. Thus, when CAR-T cells are designed to recognize a certain TAA, they attack not only tumor cells but also normal tissues expressing that antigen [199]. This challenge is particularly acute in solid tumor treatment. For example, Morgan et al. found that HER2-targeting CAR-T cells were associated with serious safety events. Within 15 min after cell infusion, the patient developed respiratory distress and eventually died after treatment. Subsequent studies established that this lethal toxicity was not attributable to off-target effects, but rather to the expression of low levels of HER2 antigen in normal lung epithelial cells, leading to significant on-target/off-tumor toxicity of CAR-T cells against healthy tissues, which resulted in severe lung inflammation and pulmonary edema. This classic case directly reveals that on-target/off-tumor toxicity is a key obstacle limiting the safe application of CAR-T cell therapy in solid tumors [200].

Beyond antigen co-expression, CAR-T cell therapy also faces additional challenges posed by the solid tumor microenvironment. The dense extracellular matrix (ECM) and abnormal vasculature create physical barriers that hinder effective T cell infiltration, limiting CAR-T cell trafficking and migration to tumors [201]. Additionally, immunosuppressive cells in the TME and key regulators such as the AP-1 family factor Basic Leucine Zipper ATF-like Transcription Factor (BATF) and thymocyte selection-associated high-mobility group (TOX) proteins induce CAR-T cell exhaustion [202, 203]. This presents a paradox: even if CAR-T cells accurately recognize tumor antigens, they often cannot infiltrate effectively or rapidly become exhausted upon entering the microenvironment.

#### Non-specificity of delivery vectors

The targeting of synthetic biology therapeutic systems is also limited by the delivery vectors themselves. Adenoviral vectors (AdVs), adeno-associated viruses (AAVs), and lentiviruses (LVs) commonly used today can be modified to efficiently deliver CRISPR-Cas9 and base editors to the nucleus, suitable for various in vivo and in vitro editing scenarios [204]. However, viral vectors possess intrinsic preferences. For instance, AAV has a strong natural tropism for liver tissue, limiting its targeted delivery efficiency to extrahepatic tissues such as solid tumors (e.g., lung and gastric cancer) [205]. HIV-1-based lentivirus tends to integrate into active transcription regions of the host genome, enabling long-term stable expression of foreign genes but also increasing the risk of insertional mutagenesis and proto-oncogene activation [206]. These vector-intrinsic properties make delivery of therapeutic genes somewhat random rather than targeted.

A more fundamental issue is that traditional viral vector strategies deliver the coding sequences for Cas9 and gRNA in the form of DNA, meaning that the gene editor is expressed in target cells for an extended period. While prolonged expression may improve editing efficiency, it proportionally increases the probability of off-target editing and immunogenicity risk, as the longevity of Cas protein expression generally favors antigen presentation and thus potential immune reactions [207, 208].

#### Improvement strategies by synthetic biology

##### High-fidelity gene editors development

Researchers have created several generations of high-fidelity SpCas9 variations to address off-target editing. Slaymaker et al. used structure-guided protein engineering to improve the specificity of Streptococcus pyogenes Cas9 (SpCas9), generating “enhanced specificity” SpCas9 (eSpCas9) variants that reduce off-target effects while maintaining robust on-target cleavage [209]. Variants like SpCas9-HF1 and eSpCas9 significantly diminish off-target activity by thousands of times while substantially maintaining on-target editing efficacy. SpCas9-HF1 maintains ≥ 70% targeting efficacy in 86% of 37 evaluated gRNAs by diminishing non-specific DNA backbone interactions via the N497A/R661A/Q695A/Q926A quadruple mutations, resulting in nearly total eradication of genome-wide off-target occurrences. eSpCas9(1.1) diminishes off-target sites by over 90% while preserving wild-type editing efficiency at the majority of sites by neutralizing positive charges in the binding pockets of non-complementary strands (K848A/K1003A/R1060A) [209, 210]. Cromer et al. further showd that high-fidelity Cas9 can be delivered to primary human hematopoietic stem and progenitor cells under clinically relevant conditions without introducing or enriching tumorigenic variants [211].

##### Logic-gated CAR design

To address on-target/off-tumor toxicity, synthetic biology provides the concept of logic gating. A classic design is the AND gate CAR, where T cells require simultaneous recognition of two tumor-associated antigens for full activation [212]. A representative technology platform is the SynNotch receptor system: recognition of the first antigen initiates CAR expression, and then the CAR recognizes the second antigen to activate T cells against cells that co-express both TAAs. This “double-lock, double-control” mechanism greatly reduces the risk of on-target/off-tumor toxicity [213].

##### Breakthroughs in novel delivery systems

At the delivery level, Engineered Nucleocytosolic Vehicles for Loading of Programmable Editors (ENVLPE) technology represents an important breakthrough direction. Unlike traditional viral vectors that rely on coding sequence transduction, ENVLPE uses fully assembled Cas9 ribonucleoprotein (RNP) as the core cargo, and achieves efficient enrichment and precise packaging of functional RNP by reprogramming lentiviral Gag protein with nuclear-cytoplasmic shuttling signals (NLS-NES) and PCP-PP7 RNA aptamer systems. This strategy completely avoids the risk of DNA integration, delivers the editor in a transient, exogenous DNA-free manner, and combined with Csy4-mediated pegRNA protection, shows high editing efficiency and low off-target characteristics in primary T cells, hematopoietic stem cells, and in vivo models. This provides a safe and efficient delivery platform for clinical translation of high-fidelity CRISPR and CAR-T logic gating. In cell therapy, ENVLPE modifies MHC I on T cells via gene editing, and it has been observed that ENVLPE-treated T cells are not rejected in recipients, opening new possibilities for developing “off-the-shelf” allogeneic CAR-T therapy [214].

##### Engineered bacteria as targeted delivery platforms

Alongside viral and synthetic particle vectors, modified bacteria are emerging as a novel paradigm for improved targeting as active delivery vectors. Employing synthetic biology techniques, modified bacteria can be meticulously designed to inhabit particular tissue microenvironments, such as hypoxic tumor areas, and deliver therapeutic agents in situ [215]. The implementation of several biosafety techniques, including suicide gene switches, auxotrophy designs, and toxin-antitoxin (TA) systems, enhances the safety and controllability of these living vectors in vivo [216–218].

The lack of in vivo editing targeting is a fundamental challenge for clinical application of synthetic biology. The collaborative development of high-fidelity editors [219], logic-gated designs, ENVLPE-like transient delivery systems [214], and engineered bacterial delivery platforms [214] is providing multi-level, complementary solutions. However, most of these strategies are still in preclinical or early clinical stages, and their long-term safety and efficacy need verification in larger human trials.

### Cost and stability of medical applications

The absence of targeting constitutes the initial obstacle in the clinical translation of synthetic biology, while exorbitant costs and system instability represent the subsequent challenges that hinder large-scale implementation [220, 221]. The former pertains to affordability, whereas the latter relates to reliability.

#### Production costs and universal therapy strategy

The R&D and production of synthetic biology technologies require substantial capital investment, including intellectual property, experimental equipment, raw materials, and production facilities. According to industry analyses, high costs remain a major factor restricting commercialization [222]. The severity of this problem is particularly prominent in cell therapy. Taking autologous CAR-T therapy as an example, the cost of goods (COGS) per batch is estimated to exceed $16,000 [223]. The underlying reason for the high cost is that CAR-T therapy is more like a customized medical service than a standardized industrial product.

The fundamental solution to the cost problem is to shift from a service model to a product model. The allogeneic cell therapy offers a distinct economic advantage over autologous approaches, as a single manufacturing campaign from a healthy donor can generate lot-sizes capable of meeting commercial demands for large patient numbers, thereby reducing the cost of goods per dose through economies of scale [224]. It has been estimated that microbial platforms can reduce manufacturing costs by 70%−90% compared to traditional methods [128]. However, allogeneic transplantation strategies also carry risks. The main challenge is that allogeneic cells may be rejected by the recipient's immune system [225]. To address this challenge, researchers are exploring various genetic engineering strategies. Deuse et al. used CRISPR to knock out MHC class I molecules to reduce immunogenicity, or overexpress “don’t eat me” signals such as CD47 to evade immune surveillance [225]. Carlsson et al. initiated a clinical trial of allogeneic islet cell therapy for type 1 diabetes, using CRISPR-Cas12b gene editing combined with lentiviral transduction to modify cells and prevent rejection, thereby eliminating the need for immunosuppressive drugs; preliminary data indicate that the therapy is effective [226].

#### Functional instability of gene circuits

The instability of gene circuits is the most stubborn technical bottleneck. This instability manifests at three levels: evolutionary degradation, host-circuit crosstalk, and dynamic behavioral drift [227–229].

##### Evolutionary degradation

When synthetic gene circuits are integrated into host cells, mutant cells that fail to express or poorly express the circuit gain a growth advantage, spread throughout the population, and ultimately lose engineered functionality [230]. Byrom and Darlington systematically revealed the seriousness of this problem: functional degradation of engineered gene circuits due to mutation and selection is a fundamental obstacle limiting long-term application. They developed a genetic controller that extends the functional half-life of circuits by almost thrice via negative feedback regulation [227]. Another breakthrough is the AI-guided gene fusion strategy STABLES, which links target genes to essential endogenous genes, providing a general stabilization framework independent of host and gene [220].

##### Host-circuit crosstalk

Gene circuits exist within living cells that contain their own inherent regulatory networks. Growth-mediated feedback between synthetic gene circuits and host organisms leads to diverse emerged behaviors, as circuit expression inevitably causes metabolic burden to host cells and thus affects cell growth, which in turn changes gene expression of circuits. This bidirectional interaction can attenuate the desired functions of gene circuits in a network topology-dependent manner [229].

##### Stability of delivery vectors themselves

The inverted terminal repeat (ITR) sequences in AAV plasmids are prone to partial or complete deletion during bacterial amplification, a known technical difficulty in AAV production [231]. Similarly, maintaining sequence accuracy of DNA templates and integrity of post-transcribed mRNA in mRNA therapy directly determines the success of downstream experiments and clinical applications [232].

### Safety barriers in the human clinical trials

#### Immune system barriers

The immune system barrier is the most direct and fundamental safety challenge faced by synthetic biology therapeutics after entering the body. It manifests mainly in three aspects: immunogenicity of exogenous proteins, CAR-T cell toxicity, and biosafety risks from engineered microorganisms.

Immunogenicity is a core bottleneck. Most synthetic biology tools contain protein sequences not encoded by the human genome, including CRISPR/Cas9 nucleases and the single-chain variable region of CARs. This means that when these exogenous proteins are introduced into the body, the immune system naturally recognizes them as invaders and mounts an attack [233, 234]. Najera et al. found that after delivery of the Staphylococcus aureus Cas9 gene by AAV2, cells presented a highly conserved SaCas9-derived T cell epitope that effectively activated CD8⁺ T cells and induced killing of transduced cells [235]. This finding has important clinical implications: even the catalytic domain of Cas9 can contain epitopes recognizable by the immune system. Moreover, the AAV vector itself elicits an immune response; AAV capsid proteins can be recognized by pre-existing or treatment-induced anti-AAV neutralizing antibodies (NAbs), resulting in reduced transduction efficiency or treatment failure [236]. These immunological barriers pose a particularly severe challenge for indications requiring prolonged or repeated dosing.

CAR-T cell treatment represents a highly effective implementation of synthetic biology in immunotherapy, exhibiting the most distinct and extensive toxicity profile. The predominant and potentially fatal toxicities include cytokine release syndrome (CRS) and neurotoxicity, immune effector cell-associated neurotoxicity syndrome (ICANS). CRS is caused by massive release of pro-inflammatory cytokines after CAR-T cell activation; clinical manifestations range from fever and hypotension to multiple organ failure. ICANS presents with symptoms from aphasia and confusion to cerebral edema and seizures. Although CRS and ICANS are often managed with tocilizumab and glucocorticoids, high-grade toxicity can still be fatal [237]. Additionally, clinical experience reveals more complex late toxicities. Serious side effects appear weeks or months after CAR-T therapy, such as late neurotoxicity and intestinal inflammation in multiple myeloma patients, which share a common immunological root and are significantly associated with non-cancer mortality [238]. Synthetic biology has offered numerous solutions to these toxicity issues. The iC9 suicide switch can trigger CAR-T cell death in cases of acute toxicity, serving as an emergency measure [239]. Logic gating requiring double antigen recognition through an “AND gate” reduces the probability of false activation from the outset [240]. Small-molecule regulatory systems allow on-demand control of CAR-T cell activity during treatment [241]. These strategies have shown good safety and controllability in preclinical models.

Microbial therapy platforms represented by engineered bacteria have attracted attention for their unique advantages in tumor-targeted delivery and metabolic disease regulation. However, such “living” properties bring biosafety problems not seen with traditional medicine. The primary risk is horizontal transfer of genetic material. Synthetic gene circuits carried by engineered bacteria may spread to other wild microorganisms in complex microbial communities through conjugation, transduction, or transformation, leading to unpredictable ecological consequences [242]. Although most engineered bacteria are designed to be auxotrophic or equipped with suicide circuits, the actual containment effectiveness and operational stability of these biosafety measures in complex natural microbial communities still lack systematic verification [242]. At the same time, the risk of environmental release must not be overlooked. Although modified bacteria are intended to inhabit the body, they may nevertheless be released into the external world via excreta. Huang et al. have examined the possible biosafety hazards associated with the release of synthetic biology microbes into drinking water in industrial environments [243] (Table 4).

**Table 4:** Key targeting and safety limitations in synthetic biology-based biomedical applications

Core application	Primary limitation category	Sub-mechanism	Specific description	Key data/outcome	Refs
ERBB2‑targeted CAR‑T for metastatic colon cancer	Safety hurdle in human trials	Fatal cytokine storm	Within 15 min after infusion: respiratory distress, sharp rise in cytokines, multi‑organ failure	Patient died on day 5; peak serum IL‑6 = 34,467 pg/mL	[200]
CAR‑T for solid tumours	delivery non‑specificity	Infiltration barrier (abnormal vasculature, dense ECM, immunosuppressive cells)	Abnormal vessels and dense ECM physically block CAR‑T entry; Treg/MDSC/TAM exclude CAR‑T	Combination strategies (vascular normalisation, matrix degradation) are still under development	[201]
HIV‑1/lentiviral vector integration analysis	delivery non‑specificity	Integration bias into active genes and hotspots	Lentiviral integration strongly favours active genes (69% in transcription units) and local hotspots (2.5 kb harbouring 1% of sites)	In vitro random integration shows no such bias, confirming in vivo targeting deviation	[206]
In vivo CRISPR‑Cas9 gene therapy	CRISPR off‑target	Off‑target cleavage and low HDR efficiency	Cas9 can cut at unintended sites; homology‑directed repair is inefficient, especially in non‑dividing cells	Genome‑wide methods (e.g. Digenome‑seq) are required to detect off‑target effects	[207]
AAV vector gene therapy	Safety hurdle in human trials	Pre-existing and induced neutralising antibodies (NAbs)	Pre-existing NAbs to AAV; post-dosing NAbs increase > 10,000-fold	AAV5 may be less sensitive to low-titre NAbs, but immune exclusion remains a major obstacle	[236]
BCMA-targeted CAR-T for multiple myeloma	Safety hurdle in human trials	Cytokine storm/infection	High non-relapse mortality (NRM) and severe infections due to cumulative high-dose steroids	1-year NRM ≈17%; 60% of NRM deaths due to infections; 5 deaths attributed to CirAEs	[238]
Detection of horizontal gene transfer in microbial communities	delivery non‑specificity	Non‑specific host range of MGEs (plasmids, phages)	Mobile genetic elements exhibit broad host ranges, making specific strain targeting difficult	CRISPR arrays can record exposure, but delivery specificity remains a bottleneck	[242]
In vivo CRISPR-Cas9 generation of lung cancer model	CRISPR off-target	Non-expected rearrangements (deletions, translocations)	Cas9 induces large deletions and unintended translocations besides the desired inversion; efficiency ~3–4%	Successful Eml4-Alk model, but diverse off-target products are generated	[244]
CRISPR‑Cas9 engineering of human T cells (CAR‑T)	CRISPR off‑target	Chromosome loss	Cas9 editing leads to whole or partial chromosome loss in 5‑20% of cells depending on gRNA	Chromosome‑lost T cells persist for weeks in culture and show a proliferative disadvantage	[245]
In vivo AAV gene therapy (mouse tropism atlas)	delivery non‑specificity	Serotype‑dependent tropism	10 AAV serotypes exhibit vastly different tropism across 22 mouse tissues; precise targeting is difficult	AAV4 transduces > 85% of lung endothelial cells but shows very poor liver transduction	[246]

#### Ethical barriers

The ethical barriers facing synthetic biology therapy in human clinical trials focus on three dimensions: the scope of technology application, dignity and value of life, and ecology and social equity. The lack of clear, unified solutions to related disputes has become an important factor restricting clinical translation.

The core focus of ethical disputes is the ambiguity of technology application scope. Synthetic biology takes the engineering and modification of living organisms as its core logic, treating life as an object that can be designed, edited, and reconstructed, which gradually erodes the inherent boundaries of traditional concepts of life. This human intervention and remodeling of life forms continuously impact existing ethical cognition and value frameworks. Around key issues such as the reasonable extent of life modification and the moral bottom line of human intervention, the academic community has not yet formed a unified consensus nor established a perfect, uniform ethical standard system, profoundly restricting clinical application and development of related technologies.

The demarcation of the ethical boundary between human germline and embryo editing is an even more sensitive issue. When synthetic biology technology is applied to germ cells or embryos, its modifications can be passed to future generations, potentially altering the human gene pool and touching core ethical bottom lines of human dignity, intergenerational equity, and species integrity. Currently, there are significant global differences in ethical norms for germline editing. Some countries explicitly prohibit germline gene editing, while others allow it only for scientific research purposes [247].

In addition, synthetic biology therapy encounters challenges related to ecological ethics and social equality. From an ecological ethics perspective, the colonization of engineered microorganisms within hosts and their potential release into the environment may lead to ecosystem disruption, extinction of native species, horizontal gene transfer, and unforeseeable ecological risks [243]. Consequently, reconciling technological implementation with environmental conservation has emerged as a crucial aspect of ethical governance [248]. At the level of social equity, the high R&D costs of synthetic biology therapy often translate into expensive clinical applications, potentially leading to a “genetic divide,” exacerbating unequal distribution of medical resources, and violating the ethical principle of medical equity. These challenges further highlight the complexity and necessity of ethical governance for synthetic biology. Balancing ecological ethical constraints with the benefits of medical resources is an inevitable prerequisite for the healthy development of synthetic biology-based diagnostic and therapeutic technologies.

#### Obstacles in regulatory system

The rapid development of synthetic biology technology far exceeds the update speed of existing regulatory systems. The lagging regulatory framework, lack of regulatory standards, global regulatory fragmentation, and insufficient adaptation to the “novelty” of the technology all constitute regulatory obstacles to clinical translation, seriously affecting technology approval efficiency and large-scale application [249].

The core dilemma is a lagging and disconnected regulatory framework. Taking the United States as an example, a report by Rice University’s Baker Institute points out that the U.S. employs a “coordinated framework” to regulate synthetic biology products: USDA is responsible for plant risk assessment, Environmental Protection Agency (EPA) for environmental applications, and Food and Drug Administration (FDA) for medical and food products [250]. However, when this framework was established, the complexity and uncertainty of synthetic biology technology meant that relevant laws and regulations could not fully address the various risks arising from commercialization of synthetic biology products. At the same time, the unpredictability of logic operations and feedback control in gene circuits makes their behavior “emergent”, exceeding the scope of traditional regulatory evaluation of “deterministic” products, leading to a growing regulatory gap [251]. The Baker Institute report recommends that researchers plan compliance pathways in advance through data submission, public engagement, and pre-review meetings to facilitate R&D translation [250].

Lack of regulatory standards further exacerbates translation difficulty. Synthetic biology therapeutic products are characterized by “personalization, living nature, and programmability”, fundamentally different from traditional chemical and biological drugs. Currently, there are no unified global standards for quality control, safety evaluation, and long-term risk monitoring. It is worth noting that regulatory agencies are actively responding to this challenge. In 2025, the FDA suggested a novel “rational mechanism” approval pathway designed to expedite the approval of personalized gene treatments and other customized medications for rare diseases when the biological etiology is established [252]. In May 2025, the inaugural patient globally received a tailored CRISPR therapy derived from this novel pathway [253], signifying the initiation of regulatory systems striving to establish specialized channels for personalized, highly customized synthetic biology treatments, and offering guidance for future iterative enhancements of regulatory frameworks.

## Future prospect of synthetic biology in biomedicine

### Precision and intelligence

In the future, synthetic biology will transcend the existing constraints of single-logic gene circuit design and concentrate on creating intricate synthetic circuit systems featuring multi-input, multi-output, and negative feedback regulation. Multiple synthetic gene circuits can sense distinct inputs, compute the magnitude of the response (“program”) according to the desired input–output relationship, and implement such response [254]. Targeting the characteristics of lesion microenvironments in different diseases such as tumors, inflammation, and metabolic disorders, it will accurately identify specific markers (e.g., hypoxic state in the tumor microenvironment, acidic pH [255], specific tumor antigens, reactive oxygen species (ROS) enrichment, arthritic inflammatory factors such as TNF-α and IL-6, abnormal metabolites in metabolic disorders), and construct logic gate systems with multiple signal responses (e.g., AND gate, OR gate [255], NOT gate [256]). The core advantage of such complex logic circuits is that only when all pathological signals are present simultaneously and reach a specific threshold will the therapeutic module be accurately activated [255], initiating targeted functions such as cell killing, therapeutic drug release, and immune regulation, thereby eliminating non-specific damage to normal tissue cells from the outset and minimizing side effects.

Simultaneously, to enhance treatment safety and controllability, synthetic biology will integrate diverse external regulatory methods, including light modulation, temperature regulation, ultrasound application, and small-molecule induction [169], facilitating real-time, non-invasive, and reversible management of the treatment process. Even in the complex in vivo physiological environment, therapeutic functions can be flexibly turned on or off, and treatment intensity dynamically adjusted according to changes in the patient’s condition, avoiding over-or under-treatment. In addition, intelligent closed-loop theranostic systems are an important direction for future synthetic biology development [257], making biomedical intervention more precise, intelligent, efficient, and controllable.

### Universal strategy and off-the-shelf

In the future, synthetic biology will fully promote the R&D and industrialization of universal cell therapy technologies such as UCAR-immune cells, completely breaking free from the limitations of autologous preparation, and realizing universal, off-the-shelf, and low-cost, thereby enabling more patients to benefit from this advanced treatment technology. The fundamental technology of universal cell therapy involves the systematic modification of cells from healthy donors utilizing the integration of synthetic biology and precise gene editing techniques. This method facilitates the development of a universal cell chassis that is non-immunogenic and suitable for standardized large-scale manufacture [258].

Naeem et al. modified healthy donor-derived T cells by deleting HLA-I molecules or overexpressing immunosuppressive molecules (such as HLA-E and HLA-G), to avoid recognition by the host immune system [259]. Introduction of CAR structures targeting specific tumor antigens and safety suicide switches (e.g., iCasp9, HSV-TK, etc.) [260] allows rapid induction of apoptosis in UCAR-T cells via small-molecule drugs in the event of serious adverse reactions, ensuring clinical safety. In addition to UCAR-T cells, synthetic biology will also promote the development of universal CAR-NK cells [261], induced pluripotent stem cell (iPSC)-derived macrophages [262], and mesenchymal stem cells (MSCs) [263], forming a diversified matrix of off-the-shelf cell products to cover treatment needs for different diseases. On this basis, a standardized universal cell bank will be established, and large-scale production of UCAR-immune cells is achieved through industrial-scale culture, rigorous quality control, and cryopreservation [264].

### Deep integration of multiple disciplines

The profound integration of artificial intelligence and synthetic biology facilitates a transition from experience-based to data-driven and intelligent design. The use of AI technologies, including big data analysis, deep learning, and algorithmic modeling, enables the integration of extensive multi-omics data (genome, metabolome, and single-cell omics) with synthetic biology experimental data to develop precise prediction models [265]. In the future, synthetic biology will be able to achieve precise design of functional elements (e.g., promoters and regulatory proteins), rational optimization of gene circuits, and personalized customization of cell engineering programs [266, 267]. AI can also predict therapeutic effects, toxicities, and pharmacokinetic characteristics of synthetic biology therapies in vivo, providing a scientific basis for clinical protocol formulation and avoiding potential treatment risks [268].

The integration of materials science and synthetic biology can provide high-quality delivery vectors and microenvironmental scaffolds for synthetic biology therapies to further optimize therapeutic effects. Through the development of smart responsive biomaterials, targeted delivery and sustained release modulation of therapeutic agents can be realized [269, 270]. Using nanomaterials to modify delivery carriers can enhance targeting and cellular entry efficiency, improving the in vivo performance of therapeutic agents [271].

In addition, data science, nanotechnology, physics, and other disciplines will also provide important support for synthetic biology development [272]. Driven by deep interdisciplinary integration, an intelligent theranostic platform integrating precise diagnosis, personalized treatment, dynamic monitoring, and real-time regulation will be created in the future.

### Expanding the spectrum of diseases

Currently, the utilization of synthetic biology in biomedicine is primarily concentrated on tumor therapy. In the future, as advancements in synthetic biology technologies progress, interdisciplinary integration intensifies, and clinical translation systems enhance, synthetic biology will perpetually expand its applications in biomedicine.

For example, in the case of infectious diseases, for bacterial, viral, fungal, and other pathogen infections, especially for drug-resistant bacterial infections and latent viral infections, synthetic biology will provide new treatment solutions that are non-antibiotic-dependent, precisely targeted, broad-spectrum, and efficient. For instance, Alfageme-Abello et al. have engineered T cells that can accurately identify and eradicate cells harboring latent HIV infection, aiming for a functional cure [273]; they have also developed probiotics designed to target and colonize the gut or infection sites, secrete antimicrobial peptides, selectively eliminate drug-resistant bacteria, preserve normal gut flora, and prevent antibiotic-induced dysbiosis and superinfection [274].

For autoimmune diseases, synthetic biology will enable precise management of immunological balance in rheumatoid arthritis, systemic lupus erythematosus, type 1 diabetes, multiple sclerosis, and other disorders through the use of artificially constructed synthetic regulatory circuits. For example, engineered cells or living therapeutic agents selectively release anti-inflammatory factors only at inflammatory lesions to re-establish immune homeostasis, thereby protecting tissues from immune attack without inducing systemic immunosuppression [275].

Moreover, for metabolic diseases such as diabetes, obesity, and non-alcoholic fatty liver disease, engineering and modifying gut microorganisms, hepatocytes, and islet cells can reconstitute metabolic pathways and achieve precise regulation of blood glucose and blood lipids, replacing long-term dependence on traditional drugs [276]. For Alzheimer’s disease, Parkinson’s disease, spinal cord injury, and other neurodegenerative diseases or nerve injuries, engineered neural stem cells, exosomes, and other carriers can be used for precise delivery to brain lesion sites, secreting neurotrophic factors, repairing damaged neurons, reconstructing neural circuits, and achieving repair and improvement of nerve function [277].

### Concluding remarks

**Fig. 4:**
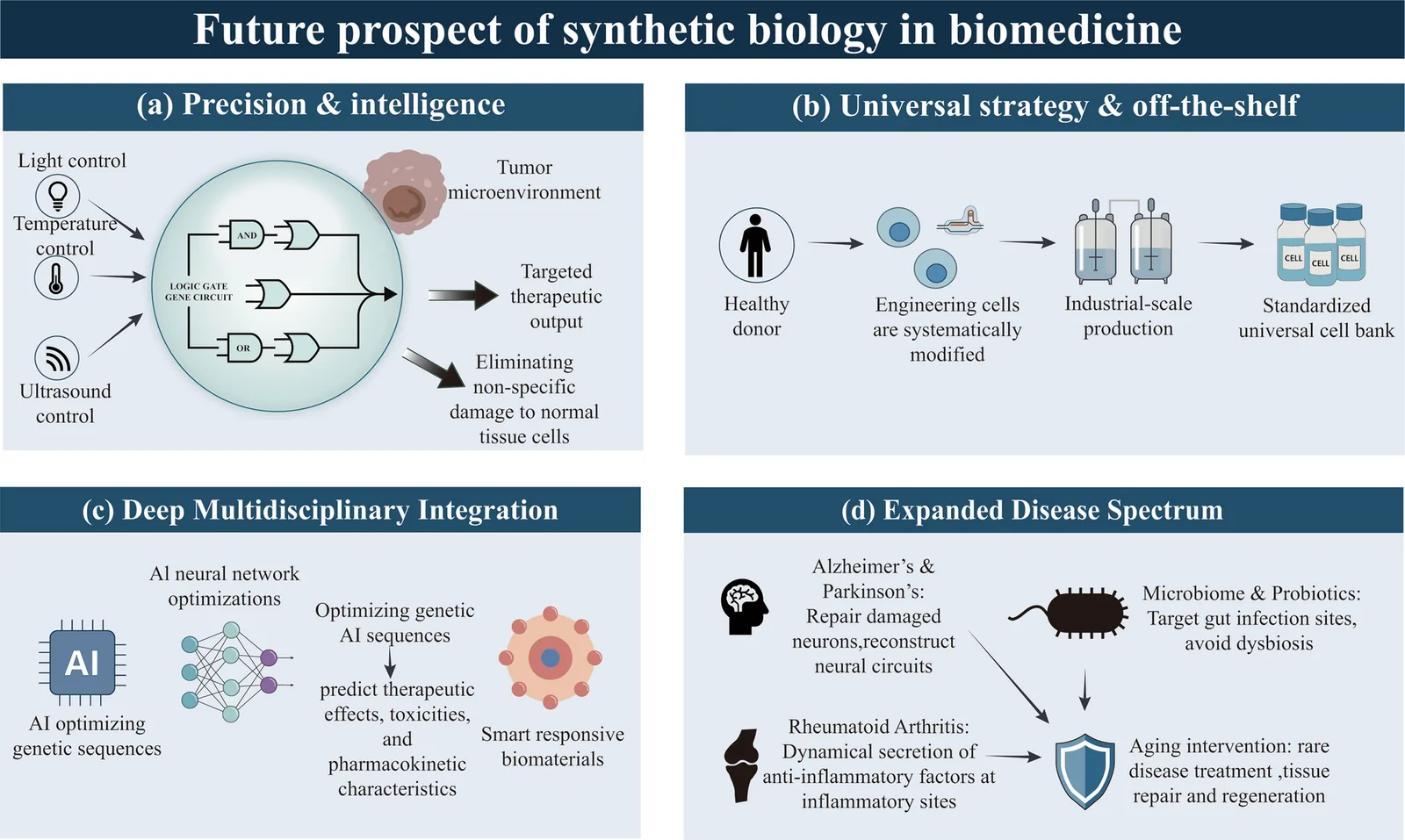
Future prospect of synthetic biology in biomedicine. This figure illustrates four core developmental orientations of synthetic biology for biomedical applications. **a** Precision & intelligence: intelligent precision control supports targeted therapeutic outputs. **b** Universal strategy & off‑the‑shelf: standardized universal cell manufacturing promotes clinical translation. **c** Deep Multidisciplinary Integration: multidisciplinary integration with artificial intelligence optimizes genetic engineering and biomaterial design. **d** Expanded Disease Spectrum: synthetic biology approaches expand therapeutic options for neurodegenerative, autoimmune, infectious and aging‑associated diseases. Collectively, these directions are jointly driving the clinical advancement of this discipline

In summary, synthetic biology is transitioning from a specialized engineering discipline into a broadly applicable medical platform. The convergence of intelligent gene circuit design, standardized cell manufacturing, AI-driven optimization, and expanding disease applications will collectively redefine how diseases are diagnosed, treated, and prevented. As these trajectories converge, synthetic biology holds the potential to deliver therapies that are not only more precise and accessible but also adaptable to the complex and dynamic nature of human disease, marking a fundamental shift in the practice of modern medicine. In addition, synthetic biology will gradually expand to aging intervention [278], rare disease treatment, tissue repair, and regeneration. With the comprehensive expansion of the disease spectrum, synthetic biology will no longer be limited to treating a single disease but will cover the entire process of disease prevention, diagnosis, treatment, rehabilitation, and anti-aging, becoming an indispensable core support technology in future biomedicine and providing all-round protection for human health (Fig. 4).

## Data Availability

Not applicable.
